# Cohort Profile Update: COVIDMENT: COVID-19 cohorts on mental health across six nations

**DOI:** 10.1093/ije/dyag233

**Published:** 2026-10-26

**Authors:** Helga Ask, Johanne Hagen Pettersen, Mary M Barker, Lea Arregui Nordahl Christoffersen, Sverre Urnes Johnson, Kadri Kõiv, Ingibjörg Magnusdottir, Hannah Milbourn, Anders B Nygaard, Ida Scheel Rasmussen, Thor Aspelund, Sonja Hellegjerde Brunvoll, Archie Campbell, Ida Henriette Caspersen, John Arne Dahl, Christian Erikstrup, Stephanie Le Hellard, Anikó Lovik, Arna Hauksdóttir, Hilde Heiro, Asle Hoffart, Bitten Aagaard Jensen, Anna K Kähler, Karl Trygve Kalleberg, Shiyu Li, Christian Madsen, Gunhild Alvik Nyborg, Sisse Rye Ostrowski, Rachel Elizabeth Ramsey, Reedik Mägi, Erik Sørensen, Edda Bjork Thordardottir, Mie Topholm-Bruun, Nicoline Uglebjerg, Tian Xiao, Arne Søraas, Kelli Lehto, Fang Fang, Ole Birger Pedersen, Ole A Andreassen, Omid V Ebrahimi, Ingunn Olea Lund, Katrine Strandberg-Larsen, Ragnhild Brandlistuen, Unnur A Valdimarsdóttir

**Affiliations:** PsychGen Centre for Genetic Epidemiology and Mental Health, Norwegian Institute of Public Health, Oslo, Norway; Department of Child Health and Development, Norwegian Institute of Public Health, Oslo, Norway; PsychGen Centre for Genetic Epidemiology and Mental Health, Norwegian Institute of Public Health, Oslo, Norway; Centre for Precision Psychiatry, Institute of Clinical Medicine, University of Oslo, Oslo, Norway; Institute of Environmental Medicine, Karolinska Institutet, Stockholm, Sweden; Psychiatric Centre Copenhagen, Bispebjerg and Frederiksberg Hospital, Copenhagen University Hospital, Copenhagen, Denmark; Institute of Biological Psychiatry, Mental Health Services, Amager and Hvidovre Hospital, Copenhagen University Hospital, Copenhagen, Denmark; Department of Clinical Immunology, Zealand University Hospital, Køge, Denmark; Department of Psychology, University of Oslo, Oslo, Norway; Research Institute, Modum Bad Psychiatric Center, Vikersund, Norway; Estonian Genome Centre, Institute of Genomics, University of Tartu, Tartu, Estonia; Centre of Public Health Sciences, Faculty of Medicine, School of Health Sciences, University of Iceland, Reykjavik, Iceland; Centre for Genomic and Experimental Medicine, Institute of Genetics and Cancer, The University of Edinburgh, Edinburgh, United Kingdom; Department of Microbiology, Oslo University Hospital, Oslo, Norway; Section of Epidemiology, Department of Public Health, University of Copenhagen, Copenhagen, Denmark; Centre for General Practice, University of Copenhagen, Copenhagen, Denmark; Centre of Public Health Sciences, Faculty of Medicine, School of Health Sciences, University of Iceland, Reykjavik, Iceland; Department of Microbiology, Oslo University Hospital, Oslo, Norway; Institute of Basic Medical Sciences, Department of Nutrition, University of Oslo, Oslo, Norway; Centre for Genomic and Experimental Medicine, Institute of Genetics and Cancer, The University of Edinburgh, Edinburgh, United Kingdom; Centre for Fertility and Health, Norwegian Institute of Public Health, Oslo, Norway; Department of Microbiology, Oslo University Hospital, Oslo, Norway; Department of Clinical Immunology, Aarhus University Hospital, Skejby, Denmark; Department of Clinical Medicine, Faculty of Health and Medical Sciences, Aarhus University, Aarhus, Denmark; Department of Clinical Science, University of Bergen, Bergen, Norway; Institute of Environmental Medicine, Karolinska Institutet, Stockholm, Sweden; Institute of Psychology, Leiden University, Leiden, The Netherlands; Centre of Public Health Sciences, Faculty of Medicine, School of Health Sciences, University of Iceland, Reykjavik, Iceland; Section of Environmental and Occupational Medicine, Oslo University Hospital, Oslo, Norway; Department of Psychology, University of Oslo, Oslo, Norway; Research Institute, Modum Bad Psychiatric Center, Vikersund, Norway; Department of Clinical Immunology, Aalborg University Hospital, Aalborg, Denmark; Institute of Environmental Medicine, Karolinska Institutet, Stockholm, Sweden; Department of Medical Epidemiology and Biostatistics, Karolinska Institutet, Stockholm, Sweden; Age Labs AS, Oslo, Norway; Institute of Environmental Medicine, Karolinska Institutet, Stockholm, Sweden; Center for Disease Burden, Norwegian Institute of Public Health, Bergen, Norway; Department of Disease Burden, Norwegian Institute of Public Health, Bergen, Norway; Department of Microbiology, Oslo University Hospital, Oslo, Norway; Department of Clinical Immunology, Copenhagen University Hospital - Rigshospitalet, Copenhagen, Denmark; Department of Clinical Medicine, Faculty of Health and Medical Sciences, University of Copenhagen, Copenhagen, Denmark; Institute of Environmental Medicine, Karolinska Institutet, Stockholm, Sweden; Estonian Genome Centre, Institute of Genomics, University of Tartu, Tartu, Estonia; Department of Clinical Immunology, Copenhagen University Hospital - Rigshospitalet, Copenhagen, Denmark; Centre of Public Health Sciences, Faculty of Medicine, School of Health Sciences, University of Iceland, Reykjavik, Iceland; Department of Clinical Immunology, Odense University Hospital, Odense, Denmark; Section of Epidemiology, Department of Public Health, University of Copenhagen, Copenhagen, Denmark; Centre for Financial Affairs, The Capital Region of Denmark, Copenhagen, Denmark; Institute of Environmental Medicine, Karolinska Institutet, Stockholm, Sweden; Department of Microbiology, Oslo University Hospital, Oslo, Norway; Age Labs AS, Oslo, Norway; Estonian Genome Centre, Institute of Genomics, University of Tartu, Tartu, Estonia; Institute of Environmental Medicine, Karolinska Institutet, Stockholm, Sweden; Department of Clinical Immunology, Zealand University Hospital, Køge, Denmark; Department of Clinical Medicine, Faculty of Health and Medical Sciences, University of Copenhagen, Copenhagen, Denmark; Centre for Precision Psychiatry, Institute of Clinical Medicine, University of Oslo, Oslo, Norway; Department of Experimental Psychology, University of Oxford, Oxford, United Kingdom; Department of Psychiatry, University of Oxford, Oxford, United Kingdom; PsychGen Centre for Genetic Epidemiology and Mental Health, Norwegian Institute of Public Health, Oslo, Norway; Department of Child Health and Development, Norwegian Institute of Public Health, Oslo, Norway; Section of Epidemiology, Department of Public Health, University of Copenhagen, Copenhagen, Denmark; Department of Child Health and Development, Norwegian Institute of Public Health, Oslo, Norway; PROMENTA Research Center, University of Oslo, Oslo, Norway; Institute of Environmental Medicine, Karolinska Institutet, Stockholm, Sweden; Centre of Public Health Sciences, Faculty of Medicine, School of Health Sciences, University of Iceland, Reykjavik, Iceland

**Keywords:** Nordic, cohort data, registry linkages, mental health young people, post covid condition, MoBa, DNBC

Key Features

- The COVIDMENT consortium, which initially included data from 392 817 participants, aims to advance knowledge on mental morbidity before, during, and after the COVID-19 pandemic in the general population and specific risk groups.
- We expanded our scope to include young people (<26 years old), as evidence suggested they were particularly affected during the COVID-19 pandemic and high-quality data were lacking.
- We added research aims on post-infectious long-term conditions and developed a screening tool for post-COVID-19 conditions.
- Since August 2021, we have added three cohorts: the Danish National Birth Cohort, the Norwegian COVID-19 Cohort, and the Norwegian Mental Health and Adherence Project. We conducted 14 additional data collections and expanded linkage to national registries and genetic data.
- After the expansion, COVIDMENT comprises 62 694 young people (aged 14–25 years in 2020) and 306 729 adults (aged 26–100 years) who remained in the most recent data collections (May 2021–November 2025).
- For collaboration proposals, please visit our website (www.covidment.is).

## The original cohort

The original COVIDMENT consortium [1] was a large-scale research collaboration leveraging unique North European resources and infrastructures aiming to advance knowledge on mental morbidity before, during, and after the COVID-19 pandemic in the general adult population [2–7] and in specific risk groups. For example, we found that long-term risk of mental and physical morbidity was increased among persons bedridden for several days with COVID-19 [8, 9], in people with pre-existing mental health conditions [10], and in family members and friends of critically ill COVID-19 patients [11].

## What is the reason for the new focus and data collection?

The original COVIDMENT consortium primarily focused on mental health outcomes among adults during and shortly after the COVID-19 pandemic. Emerging evidence, however, shows that children, adolescents, and young adults (aged <26 years) faced distinct and significant COVID-19-related challenges, including school closures, social isolation, family stress, and disrupted access to mental healthcare [12–14]. Rates of anxiety, depression, and neurodevelopmental conditions such as Attention Deficit Hyperactivity Disorder (ADHD) have been rising among young people in recent decades [15–18], but it still remains unclear whether these trends were further accelerated by the pandemic. Therefore, we expanded the consortium’s scope to younger populations.

Post-COVID condition (PCC) is increasingly recognized and diagnosed, with persistent symptoms including general fatigue, cognitive difficulties, and mood disturbances, affecting both young and adult populations [19, 20]. These trends underscore the need for better understanding of the underlying causal factors to develop more targeted responses. Comparing PCC with other post-infectious conditions will provide critical context, helping to determine whether these outcomes are specific to COVID-19 or reflect broader post-infectious syndromes.

Continuous and adaptive data collection is essential for monitoring the long-term impacts of the COVID-19 pandemic and to prepare for future health crises. By introducing new measures, such as validated PCC screening tools, and by bringing in new waves of follow-up data, the cohorts remain responsive to emerging needs.

## What will be the new areas of research?

The updated COVIDMENT research agenda introduces two new thematic areas. The first new research area is the systematic investigation of mental health trajectories among children, adolescents, and young adults. This work extends the consortium’s original adult-focused research to developmental stages at which mental health vulnerabilities often first emerge [12–14].

Research will focus on:

- trends in mental health and well-being across the pre- and post-pandemic periods, and whether observed changes reflect pandemic-related disruptions or continuations of pre-existing trends;
- identification of risk, protective, and mechanistic factors across the pre-pandemic, pandemic, and post-pandemic periods.

Analyses will be gender-inclusive and will consider living conditions and cross-country differences. Special attention will be given to vulnerable groups, including individuals with chronic medical conditions, early-onset neurodevelopmental conditions, pre-pandemic anxiety or mood disorders, or adverse childhood experiences.

The second new research area addresses PCC and its mental and physical health consequences across age groups. By using a recently developed short screening tool, the consortium will:

- assess the prevalence, varying severity, and persistence of PCC symptoms;
- characterize mental and physical health outcomes associated with PCC across different ages;
- compare PCC with other post-infectious conditions, to assess whether mental health sequelae are COVID-specific or part of a broader pattern of post-infectious syndromes.

Both research areas will be supported by the consortium’s access to rich Nordic register data, longitudinal birth cohorts, and harmonized COVID-19 cohorts. This integrated infrastructure enables robust, population-based analyses of long-term mental health outcomes and strengthens preparedness for future public health crises.

## Who is in the cohort?

Since August 2021, when the cohort profile [1] was published, several cohorts have been added to our consortium, originally consisting of the Danish Blood Donor Study (DBDS), the Estonian Biobank (EstBB) C-19 and Mental Health Online Survey (MHoS) cohorts, the Icelandic COVID-19 National Resilience Cohort, Norwegian BRY.DEG2020, the adult COVID19 data collections from the Mother, Father, and Child Cohort study (MoBa), Scottish Generation Scotland/CovidLife, and Swedish Omtanke2020 (see Fig. 1).

**Figure 1:**
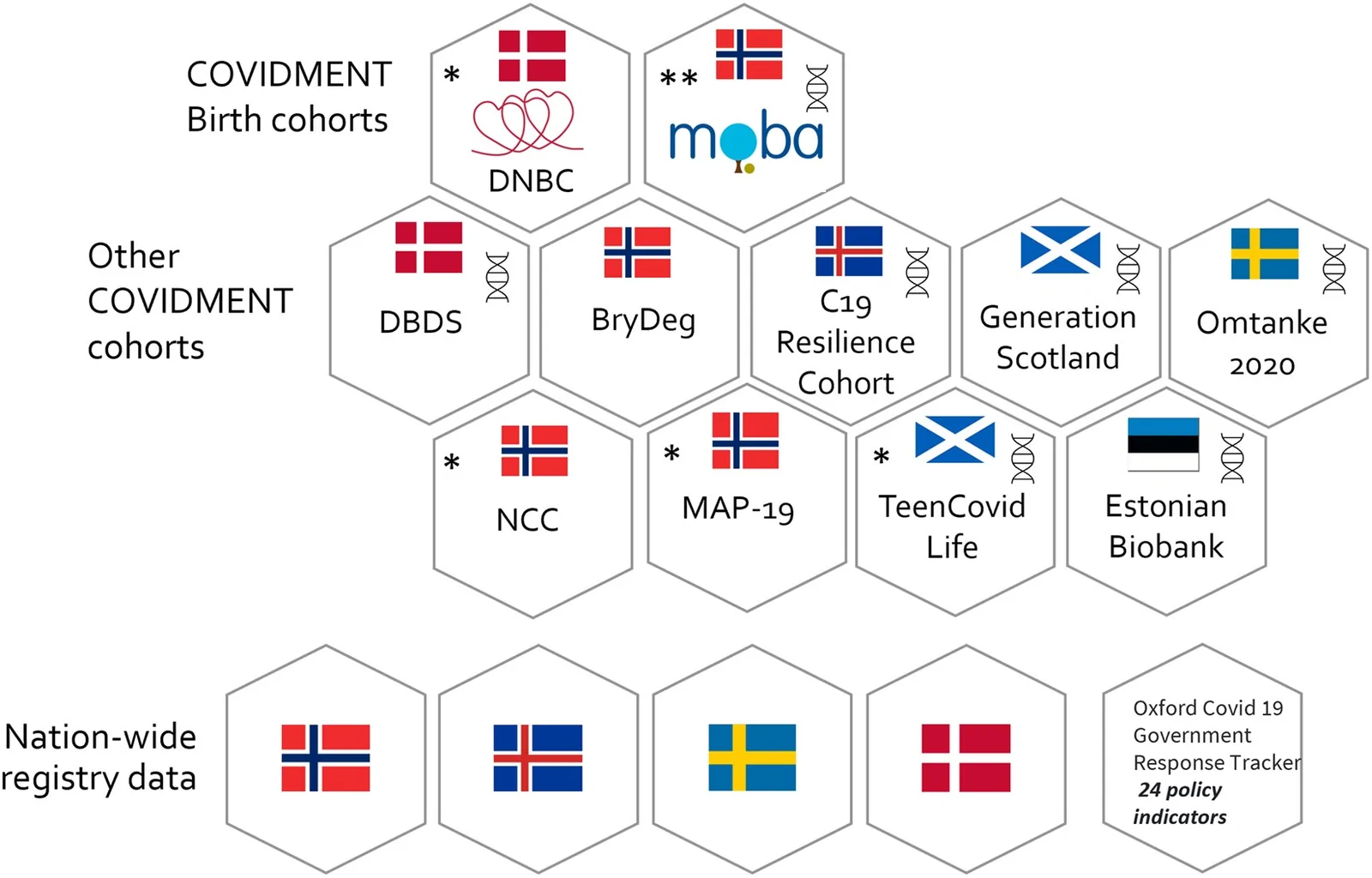
Data sources in the COVIDMENT consortium. DNBC, Danish National Birth Cohort; NCC, Norwegian COVID-19 Cohort study; MAP-19, Mental Health and Adherence Project. Health registry data have been linked across all COVIDMENT cohorts (except BryDeg).*New cohorts in this consortium update. TeenCovid Life is a separate data collection in Generation Scotland. **In the previous consortium (on adults), MoBa was only participating with the data collected on adults. For this version, we also include the data we have on the children of the birth cohort.

To enhance the focus on young people, we now include the child cohorts of two nationwide birth cohorts: the Danish National Birth Cohort (DNBC) [21, 22] and MoBa [23, 24]. In both cohorts, multiple data collections have been conducted throughout pregnancy, childhood, adolescence, and young adulthood. Some of these follow-ups were conducted in part during the pandemic (see Fig. 2).

**Figure 2:**
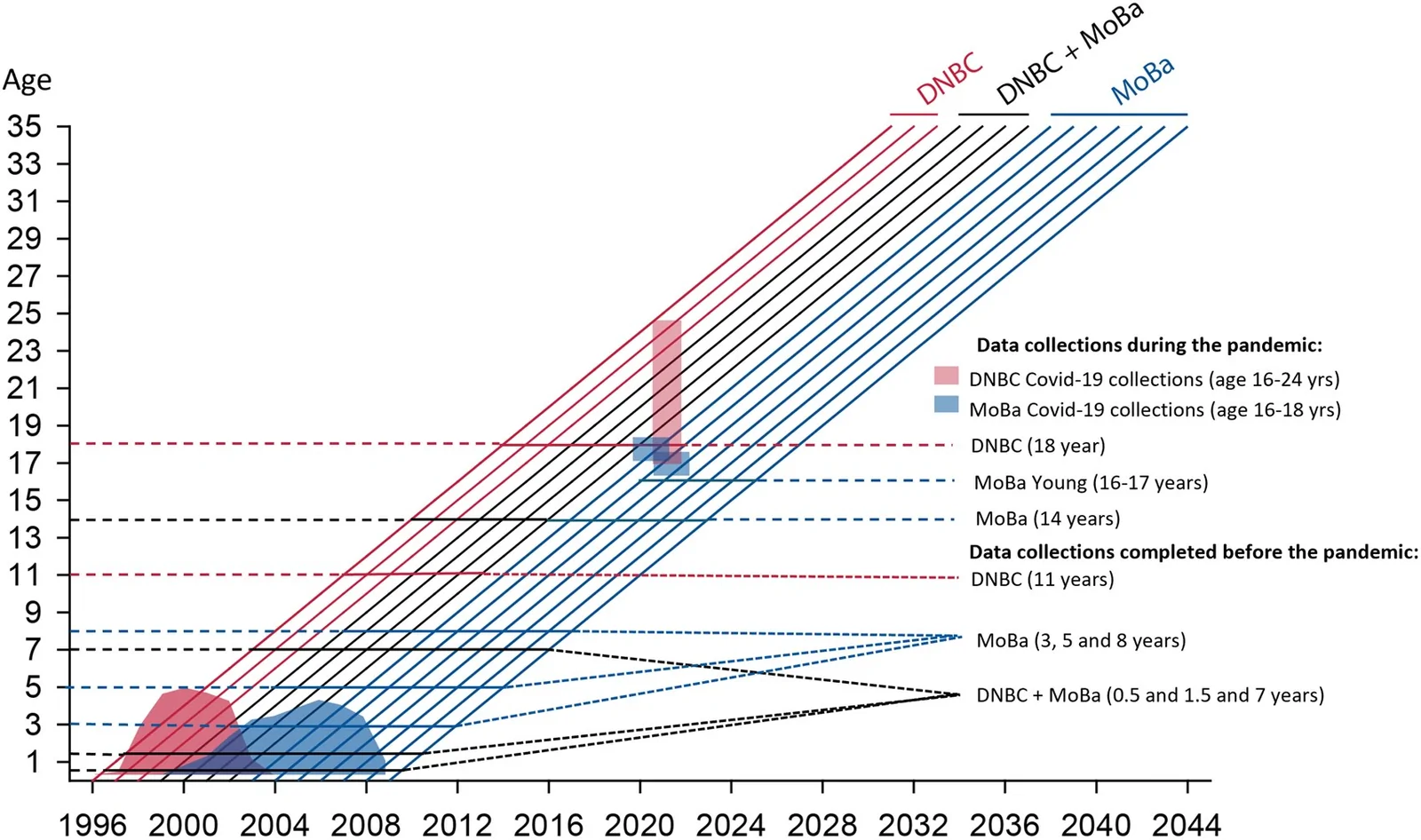
Data collections before and during the pandemic across the two pregnancy cohorts in COVIDMENT. Along the *x*-axis, the birth-year distributions of the two birth cohorts are shown (red = DNBC; blue = MoBa). Diagonal lines refer to children born within the same birth year; horizontal lines refer to data collections. In addition to the data collections illustrated here, both cohorts collected data through three questionnaires during pregnancy and one questionnaire was sent to all MoBa fathers in 2015.

DNBC COVID-19 data collections: DNBC enrolled 100 407 pregnancies in 1996–2002 (∼30% of all pregnancies in Denmark during these years). A COVID-19 data collection was launched in late March 2020, corresponding to the third week of the initial lockdown in Denmark, to examine the impact of the pandemic on everyday life, mental well-being, social isolation, compliance with precautions, and symptoms of COVID-19 [25]. In total, 53 323 adolescents aged 16–24 years were invited, with a 24% response rate for the first wave. During spring 2020, there were seven weekly waves of data collection, only inviting participants responding to previous waves. The seventh wave (May–June 2020) included 1692 adolescents (91% response rate). All participants eligible for the first wave were re-invited for a final eighth data collection, ∼1 year after the first wave. Of 54 511 invited, 31% adolescents responded. In a separate 18-year data collection, 77% of the participants completed the questionnaire before the pandemic and the rest during the pandemic. These data have been used to examine how the lockdowns influenced the young people’s mental health [26–28].

MoBa COVID-19 data collections among young people: MoBa enrolled 112 908 pregnancies in 1999–2009 (41% participation). Several youth data collections were ongoing when the pandemic came: ∼50% of mothers of 14-year-old children and the 14-year-olds themselves completed questionnaires before the pandemic and the remaining during the pandemic. In 2019, the MoBaYoung data collection was initiated, sending monthly questionnaires to 16-year-old participants. Data collections covered age-appropriate topics such as school functioning, social relationships, well-being, and mental health. For details, see the MoBa cohort profile update [24]. MoBa adolescents aged 16–17 years were also invited to complete biweekly COVID-19-specific data collections from March 2020 until April 2021—29 rounds of data collection.

We also added two new cohorts with mainly adult participants: the Norwegian Mental Health and Adherence Project (MAP-19) and the Norwegian COVID-19 Cohort study (NCC study).

MAP-19 is a longitudinal study investigating mental health and preventive health behaviours in the Norwegian general adult population during the COVID-19 pandemic and post-pandemic period [29]. The study includes 10 longitudinal assessment waves, spanning from March 2020 to March 2023. This time period covers the full duration of the pandemic-associated social-distancing policies, in addition to a 1-year follow-up after their discontinuation.

In March 2020, eligible participants (i.e. Norwegian residents aged ≥18 years providing informed consent) were recruited through an online survey disseminated to a random selection of Norwegian adults through a Facebook Business algorithm and through national, regional, and local information platforms, as described elsewhere [8, 30]. A total of 10 061 adults were enrolled, including 2908 aged 18–25 years.

The NCC study was established in March 2020 at Oslo University Hospital. Its initial aims were to investigate risk factors for the community and workplace transmission of COVID-19, and to better understand the disease and its clinical implications. Eligible participants were aged ≥18 years and had a Norwegian identification number. Participants were recruited through social media and nationwide media coverage. In addition, electronic invitations were sent to several predefined groups: people who had been tested for SARS-CoV-2 early in the pandemic, a nationally representative sub-cohort, and selected patient and occupational groups. All participants provided online informed consent (*n* = 182 560). After completing the baseline questionnaire, participants were invited to up to complete six follow-up questionnaires (50%–83% response rates). The first three were distributed at 3-month intervals, after which subsequent questionnaires were distributed at 1- to 2-year intervals (the last in March 2026).

Since the cohort profile paper was published, we have conducted 14 additional data collections across the original COVIDMENT cohorts: one in the C-19 Resilience Cohort (September–December 2022, with one also planned for 2026), two in the DBDS (spring 2021 and summer of 2022), four in Omtanke2020 (the last ending March 2025), one in BryDeg (March 2025), one in Generation Scotland (May 2022–September 2025 among newly recruited cohort participants), and five among the adult participants in MoBa (every second to sixth month, the last in November 2025). In EstBB, a new data collection was conducted in 2026.

New registry linkages and updates have been performed across the COVIDMENT cohorts (see Table 1). Of the 10 cohorts, 8 have been linked to registries containing diagnostic codes from specialist healthcare and 7 to primary healthcare registries, currently updated with data until 2022–5 across the cohorts. Nationwide registry data linkages are available across four countries (see Table 2).

**Table 1:** Healthcare registry data linkages and genetic data available across COVIDMENT cohorts.

	Denmark		Iceland	Estonia	Norway				Sweden	Scotland
	DBDS	DNBC	C19-Resilience	Estonian Biobank	BryDeg2020	MAP-19	NCC	MoBa	Omtanke2020	CovidLife
Primary healthcare			ICD-10 F codes and G47, J09-J11, T35-T50, X60-X84, Y87	ICD-10 F codes (+ all other ICD-10 codes)	–	ICPC Psychological codes	In progress (expected 2026)	ICPC Psychological codes	In progress (expected 2026)	All Read v2 codes
Specialist healthcare	ICD-8 and -10 F codes (+ most other ICD codes) + treatment of alcohol and substance use in specialized municipal clinics	ICD-10 F codes			–	ICD-10 F codes	In progress (expected 2026)	ICD-10 F codes (+ most other ICD-10 codes)	ICD-10 F codes (+ most other ICD-10 codes)	ICD-10 F codes
Prescription drugs acting on the nervous system (ATC N-codes)	All (N01–N07 + other)	All (N01–N07)	N02, N05, N06	All (N01–N07)	–	**–**	In progress (expected 2026)	All (N01–N07)	All (N01–N07)	All BNF codes
Genetic data available for analyses	X	–	X	X	–	–	–	X	–	X subset of *n* = 4358

X = data available; – = data currently unavailable in the COVIDMENT cohorts. ICPC, International Classification of Primary Care codes, Psychological codes, covering psychological symptoms and mental disorders; ICD-10, International Classification of Diseases, 10th edition; F codes, ICD codes for Mental and behavioural disorders, covering mental disorders; ATC, Anatomical Therapeutic Chemical Classification System codes; BNF, British National Formulary. DBDS was linked to the Danish Central Psychiatric Research Register in 2023, holding information on all psychiatric diagnoses given at Danish psychiatric hospitals between 1969 and 1994, and updates from the Danish National Patient Register, prolonging the follow-up until 31 December 2023, and the Danish National Prescription Register, including follow-up until June 2024. The DNBC have been linked to the same registries but up to 31 December 2023. The EstBB is regularly linked to national and regional electronic health records, including national quality registers, hospital records, the Estonian Health Insurance Fund’s database, and the National Health Information System, covering both primary and specialist care since 2004. Norwegian registry data are available for linkage covering data from 2006 (primary healthcare) and 2008 (specialist healthcare) onwards. Omtanke2020 was in 2025 linked to the Swedish Patient Register, holding information on all psychiatric diagnoses given in an inpatient hospital visit since 1973 and outpatient hospital since 2001. Furthermore, they conducted record linkage with the national health registry in 2022, providing data on psychiatric diagnoses from the Hospital Discharge Register, Register of Primary Health Care Contacts, and the Landspítali National University Hospital electronic database on emergency care, along with information on respective psychotropic drug utilization sourced from the Prescription Medicines Register. Swedish Prescribed Drug Register: prescribed medication dispensed in pharmacy since 2005.

**Table 2:** Overview of nationwide registry data on psychiatric healthcare utilization available for the total population across Denmark, Iceland, Norway, and Sweden.

	Denmark	Iceland	Norway	Sweden
**Demographic**				
Year of birth	X	X	X	X
Sex	X	X	X	X
Residential area	X	–	X	X
Educational attainment	X	–	X	X
**Mental health**				
Primary healthcare	–	ICD-10 Mental and behavioural disorders (F codes) and G47, J09–J11, T35–T50, X60– X84, and Y87	ICPC Psychological codes	ICD-10 Mental and behavioural disorders
Specialist healthcare	ICD-10 and ICD-8 Mental and behavioural disorders (F codes)		ICD-10 Mental and behavioural disorders (F codes)	ICD-10 Mental and behavioural disorders (F codes)
Prescription drugs acting on the nervous system (ATC N-codes)	All (N01–N07)	N02, N05, N06	N02A, N02B, N02C, N03A, N05A, N05B, N06A, N06B, N07B	All (N01–N07)
Cause-of-death registry	X	X	–	X
COVID-19 registry	X	X	X	X

X = data available; – = data currently unavailable in the COVIDMENT registries. For Sweden, data available are not nationwide, but restricted to the Västra Götaland and Stockholm regions; ICPC, International Classification of Primary Care codes; Psychological codes, covering psychological symptoms and mental disorders; ICD-10, International Classification of Diseases, 10th edition, covering mental disorders; ATC, Anatomical Therapeutic Chemical Classification System codes. Iceland: in early 2024, we established a linkage with nationwide registers held by the Icelandic Directorate of Health, to access comprehensive information on individuals residing in Iceland as of 1 January 2005, including birth year and gender, along with confirmed SARS-CoV-2 infections, vaccinations, and psychiatric disorders diagnosed during the pandemic. Moreover, we received data on all births or immigrations to Iceland occurring after the follow-up period, as well as details regarding the deaths or departures from Iceland.

The number of participants remaining in the 10 COVIDMENT cohorts is 306 729, including individuals from both the original and the newly added cohorts. The mean age of adult participants at the most recent data collection was 47.8 years (range 18–100 years). The timing of and participation in the most recent data collection varied substantially across cohorts (Supplementary Table 1). Some degree of healthy participant bias [31] is likely present across all 10 cohorts, as voluntary participation and continued follow-up generally favour individuals who are healthier and more socio-economically advantaged than non-participants. Evidence from the prospective pregnancy cohorts illustrates this pattern: both baseline participation (∼30%–40%) and longitudinal retention have been shown to favour older, healthier, and socio-economically advantaged women [32–36]. Such participation bias also extends to offspring who later participate independently [22, 28, 37]. Nevertheless, registry-based nationwide validation studies in both cohorts have consistently shown that, although selective participation and attrition can distort prevalence estimates and other descriptive statistics, associations between exposures and outcomes are generally robust, with little evidence of substantial bias after adjustment for key socio-demographic factors [32–34, 36–38]. Detailed assessments of selection and attrition processes have been reported separately for MoBa [32, 33, 36, 38] and the DNBC [22, 34, 35, 37, 39]. Furthermore, questionnaire non-response does not necessarily imply loss to follow-up, as most cohorts can continue participant follow-up through national registries.

## What has been measured?

The main measures were described in the Cohort Profile [1]. Participants have answered questions related to mental and physical health, occupation, and life conditions during the COVID-19 pandemic, as well as questions on COVID-19-specific factors such as symptoms and hospitalization. Details of specific cohorts can be found elsewhere (DBDS [40], Omtanke2020 [41], EstBB [42, 43]). In the NCC, subgroups of participants have been invited to answer questionnaires with an in-depth focus on topics such as cognition, diet, and health economics.

The COVIDMENT PCC screening tool has been developed through data collected from the COVIDMENT cohorts, a comprehensive review of the literature, and consultation with key stakeholders, including users, clinicians, and researchers. The tool has been included in questionnaires in Omtanke and data collection is pending in Iceland. MoBa has continuously collected PCC-relevant data (closely comparable to the screening tool) and EstBB was collecting PCC-related data in spring 2026. These data across cohorts will be combined in meta-studies.

## What has it found? Key findings and publications

To illustrate the usability of the updated consortium data, particularly from the two birth cohorts, we conducted quasi-experimental difference-in-differences (DID) analyses of outcomes related to mental health among boys and girls in MoBa and the DNBC. The impact of the COVID-19 pandemic was evaluated by comparing changes in outcomes from the pre-pandemic period to the pandemic period at follow-up. The baseline measure consisted of only pre-pandemic measures, collected at ages 8 years (Norway) and 11 years (Denmark), while outcome measures were collected from 14-years-olds (Norway) and 18-year-olds (Denmark) both before and during the pandemic. In the DID models, the general developmental age trend is separated from the changes that may be linked to the pandemic period. Each model included the main effects of time, pandemic group (pre-pandemic vs. pandemic), and their interaction (the DID estimate). Outcomes in MoBa were depressive symptoms, anxiety symptoms, ADHD traits, sleep, physical activity, and number of close friends. DNBC outcomes included self-perceived number of friends, behavioural and emotional difficulties, self-rated health, quality of life, and hours of sleep (see Supplementary methods for description). An overview of the main results is shown in Table 3, illustrated for selected outcomes in Fig. 3.

**Figure 3:**
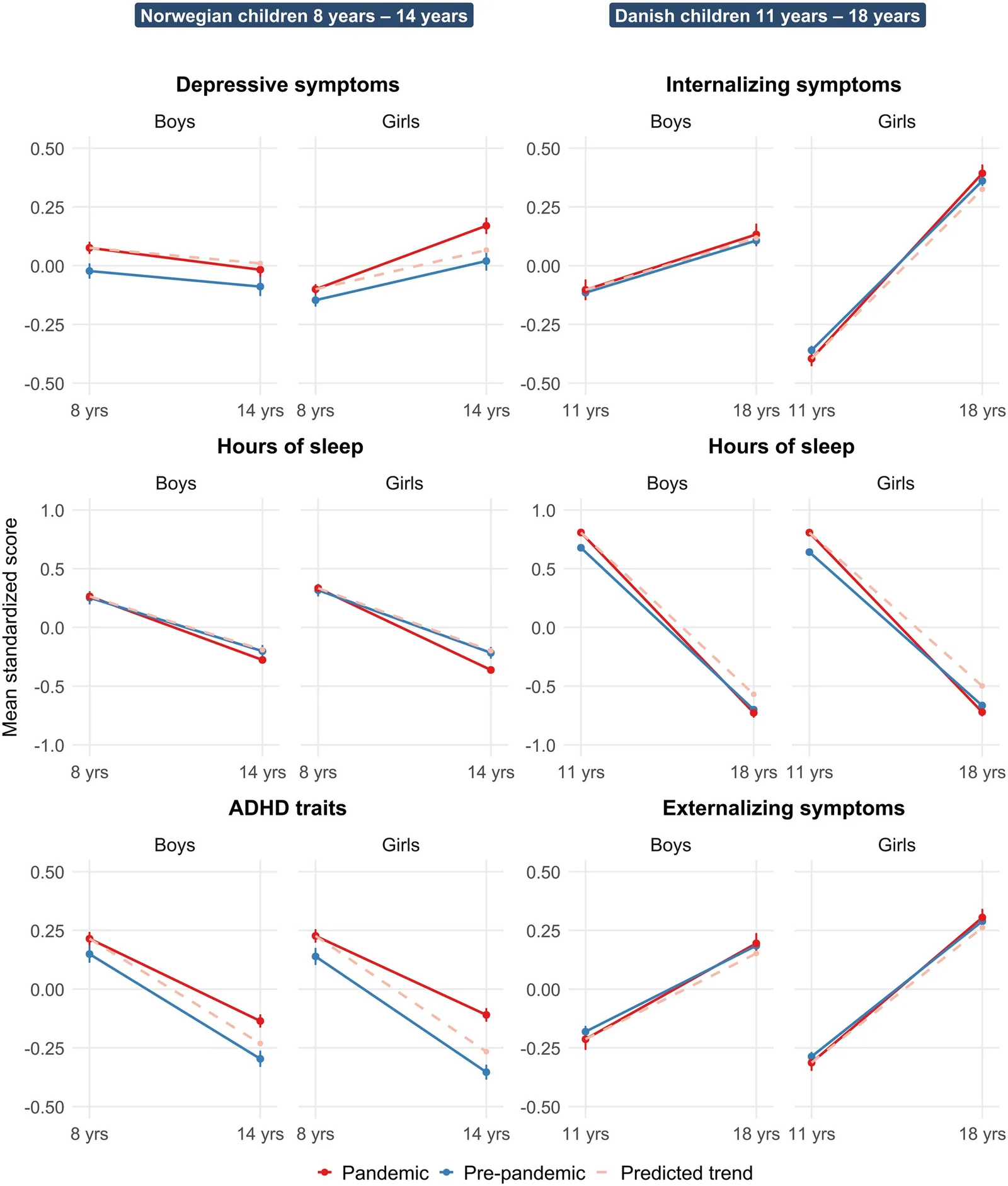
DID estimates from MoBa and the DNBC, stratified by sex. Blue lines show age-related trends among children unexposed to the pandemic (ages 8 and 14 years in MoBa; ages 11 and 18 years in DNBC). Red lines represent observed age trends among children who completed the 14-year (MoBa) or 18-year (DNBC) questionnaire during the pandemic. Dashed red lines indicate the counterfactual trajectories expected in the absence of the pandemic, under the parallel trends assumption (predicted trend). The difference between the observed and counterfactual post-pandemic trajectories corresponds to the estimated pandemic effect. Model parameter estimates are provided in the Supplementary Results.

**Table 3:** Overview of results from DID analyses across MoBa and the DNBC. Upward arrow = observed increase during the pandemic; downward arrow = observed decrease during the pandemic; dash = no change; arrows in brackets = general age trends from 8 to 14 years in MoBa and from 11 to 18 years on DNBC (independently of the pandemic). See Supplementary Results for parameter estimates for all models.

	Girls			Boys	
Measure	MoBa	DNBC		MoBa	DNBC
Depression/internalizing symptoms	↑ (↑)	↑ (↑)		− (↓)	− (↑)
Anxiety symptoms	− (↓)			− (↓)	
ADHD/externalizing symptoms	↑ (↓)	↑ (↑)		↑ (↓)	− (↑)
Sleep duration	↓ (↓)	↓ (↓)		↓ (↓)	↓ (↓)
Physical activity	↓ (↓)			− (↓)	
Number of friends	↓ (↓)	↑ (↑)		↓ (↓)	↑ (↑)
Self-rated health		− (−)			− (↓)
Quality of life		↓ (↓)			↓ (↓)
Prosocial behaviour		− (↑)			− (−)

Among MoBa females, there were pandemic effects across five of the six outcomes (DID estimates range: 0.08–0.16 fractions of SD, also persisting across sensitivity analysis; see Supplementary Results) and for five of the seven outcomes in the DNBC (DID estimates range: 0.07–0.22). Among males, pandemic effects were only observed for ADHD traits [β = 0.10, confidence interval (CI) 0.06, 0.13, *P* = 5.079 × 10^−^^7^] and reduced hours of sleep (β = −0.09, CI −0.15, −0.02, *P* = .010) in MoBa and for three of the outcomes (sleep, quality of life, and number of friends) in the DNBC (β range 0.08–0.20). The general increase or decrease in traits across development appeared to be stronger than the pandemic effect across both cohorts.

These results suggest that, while developmental changes across adolescence account for much of the variation observed in mental health and related outcomes, the pandemic period appears to have introduced an additional layer of risk, particularly for girls and for certain domains such as internalizing symptoms and sleep. The fact that these effects emerged consistently across two large population-based cohorts, with different baseline ages, measurement instruments, and national contexts, strengthens the evidence that the pandemic may have affected developmental trajectories beyond what would be expected from normative age-related changes alone.

Other key findings from the COVIDMENT consortium include:

- General population: pandemic-related changes in mental morbidity during and after the COVID-19 pandemic in the general adult population, using both self-reported [2, 4] and registry-based diagnostic codes [3].
- Specific risk groups: we found long-term mental and physical morbidity [8, 9] and impaired cognitive function [44] in persons bedridden for several days with COVID-19. Higher levels of mental morbidity were also observed among adults with prolonged quarantine exposure [7, 13], in adults with pre-existing mental health conditions [10], in children with pre-existing mental health conditions [12], and in family members and friends of critically ill COVID-19 patients [11].
- We observed links between genetic liability to psychiatric conditions and acute COVID-19 illness (and other respiratory infection) severity [45] and PCC [46].
- We observed a short-term improvement in depressive and anxiety symptoms among adults receiving COVID-19 vaccines [47].

## What are the main strengths and weaknesses?

The COVIDMENT consortium has several important strengths for studying mental health before, during, and after the COVID-19 pandemic. It brings together cohorts across multiple countries, diverse research traditions, and complementary methodological approaches, creating a strong collaborative infrastructure that has generated new funding, innovation, and continued scientific relevance beyond the acute pandemic period. This international network also provides a foundation for rapid mobilization in future societal and public health crises.

A major strength of COVIDMENT is the availability of real-time data collected during the early phases of the pandemic. These data reduce recall bias and provide direct insight into how individuals experienced rapidly changing restrictions, disruptions to daily life, and reduced access to education, work, and health services. The repeated follow-up waves further allow the investigation of mental health trajectories across the pre-pandemic, pandemic, and post-pandemic periods, enabling analyses of within-person change, identification of risk and resilience factors, and assessment of longer-term outcomes. Continued follow-up beyond the pandemic allows evaluation of whether observed mental health changes represent transient stress responses, delayed or cumulative effects, or longer-lasting shifts in population mental health.

Another key advantage is the linkage of survey data with national health registries and, in several cohorts, genetic data. This strengthens outcome ascertainment, improves follow-up completeness, and enables analyses of gene–environment interplay, vulnerability, and resilience factors. Such linkages also support more robust differentiation between pandemic-related changes in mental health and changes driven by healthcare utilization, diagnostic practices, or underlying secular trends. The inclusion of children, adolescents, and young adults, along with the development of a PCC screening tool, helps COVIDMENT address important evidence gaps in younger populations and in post-infectious health.

The consortium also has limitations. As the acute pandemic phase has ended, it is no longer possible to collect real-time data during lockdowns or rapidly changing public health measures. As in all longitudinal studies, selection bias and attrition remain concerns, and vulnerable or disadvantaged groups may be under-represented. Although the use of population-based registry data alleviates some of these problems, they capture only individuals who seek or receive healthcare and may therefore miss milder symptoms and unmet needs.

There are also analytic limitations. Current DID findings are based on a single follow-up point and cannot determine whether the observed differences persist or change over time. Average effects may mask heterogeneity, as impacts likely vary across subgroups. Disentangling pandemic-specific effects from broader pre-existing trends, particularly among young people, remains methodologically challenging and requires triangulation across survey and registry data, as well as across nations.

Going forward, COVIDMENT is well placed to use multimodal data to investigate the long-term impact of the pandemic across all age groups and increase our understanding of PCC.

## Can I get hold of the data? Where can I find out more?

We welcome proposals for collaboration; please visit our website (www.covidment.is) for further information. Data from MoBa are managed by the Norwegian Institute of Public Health. Access requires approval from the Regional Committees for Medical and Health Research Ethics (REC), compliance with General Data Protection Regulation (GDPR), and data-owner approval. Participant consent does not allow individual-level data storage in repositories or journals. Researchers seeking access for replication must apply via www.helsedata.no. Researchers can apply for the use of data from the DNBC at http://www.dnbc.dk.

## Ethics approval

Analyses based on MoBa were approved by the Regional Committees for Medical and Health Research Ethics (14140). MAP-19 was ethically approved by the Norwegian Centre for Research Data (reference: 802810) and the Regional Committee for Medical and Health Research Ethics (reference: 125510). Omtanke2020 was ethically approved by the Swedish Ethical Review Authority (reference main ethics approval: 2020–01785, as well as several ethics amendment approvals, the latest with reference 2024-06983-02). The DBDS was approved by the ethics committee [the Zealand and Central Denmark Regional Committees on Health Research Ethics (SJ-740 and 1-10-72-95-13) and the Data Protection Agency (P-2019–99)]. The activities of the EstBB are regulated by the Human Genes Research Act, which was adopted in 2000 specifically for the operations of the EstBB. Individual-level data analysis in the EstBB was carried out under ethical approval (1.1–12/1277 and 1.1–12/2860) from the Estonian Committee on Bioethics and Human Research (Estonian Ministry of Social Affairs), using data according to release applications (P09-23/07/2021 and P20-06/09/2021) from the Estonian Biobank. The NCC study was approved by the Regional Committees for Medical and Health Research Ethics of Norway (124170). The C-19 Resilience Cohort and respective linkage to nationwide registers were approved by the National Bioethics Committee, which included an evaluation from the National Data Protection Authority (NBC nrs: 20-073 and 21-071). The Committee on Biomedical Research Ethics approved the DNBC and all women who participated in the study gave informed written consent. The participants born into the DNBC were informed about their participation, rights, and how to drop out when turning 18 years old. This study was approved by the DNBC steering committee (ref. 2025-03).

## Supplementary Material

dyag233_Supplementary_Data

## References

[1] Unnarsdóttir AB , Lovik A, Fawns-Ritchie C et al Cohort profile: COVIDMENT: COVID-19 cohorts on mental health across six nations. Int J Epidemiol 2022;51:e108–e122. 10.1093/ije/dyab234 PMC8690101 35020900

[2] Lu L , Hannigan LJ, Brandlistuen RE et al Mental distress among Norwegian adults during the COVID-19 pandemic: predictors in initial response and subsequent trajectories. Int J Public Health 2023;68:1606164. 10.3389/ijph.2023.1606164 PMC10630161 38024210

[3] Jensen P , Engdahl B, Gustavson K et al Incidence rates of treated mental disorders before and during the COVID-19 pandemic—a nationwide study comparing trends in the period 2015 to 2021. BMC Psychiatry 2023;23:668. 10.1186/s12888-023-05157-1 PMC10500922 37704941

[4] Wang Y , Hauksdóttir A, Thordardottir EB et al Depressive symptom trajectories among general population during the COVID-19 pandemic in Iceland: a prospective cohort study (2020–2023). BMJ Public Health 2024;2:e001294. 10.1136/bmjph-2024-001294 PMC11816283 40018606

[5] Wang Y , Unnarsdóttir AB, Magnúsdóttir I et al Trends of perceived disruption in healthcare services during the pandemic: findings from the COVID-19 National Resilience Cohort in Iceland. Eur J Public Health 2024;34:394–401. 10.1093/eurpub/ckad220 PMC10990518 38129962

[6] Murphy GL et al The influence of social network on depressive and anxiety symptoms during the COVID-19 pandemic: findings from a Swedish cohort study. 2024. 10.1192/bjo.2025.10915 PMC12724092 41429645

[7] Lu L , Wang Y, Ebrahimi OV et al Long-term psychological effects of COVID-19-related quarantine: an observational study of three cohorts in Norway and Iceland. BMC Med 2025;23:519. 10.1186/s12916-025-04349-8 PMC12465783 40999392

[8] Magnúsdóttir I , Lovik A, Unnarsdóttir AB et al; COVIDMENT Collaboration. Acute COVID-19 severity and mental health morbidity trajectories in patient populations of six nations: an observational study. Lancet Public Health 2022;7:e406–e416. 10.1016/S2468-2667(22)00042-1 PMC8920517 35298894

[9] Shen Q et al COVID-19 illness severity and 2-year prevalence of physical symptoms: an observational study in Iceland, Sweden, Norway and Denmark. Lancet Regional Health–Europe 2023,35:100756. 10.1016/j.lanepe.2023 PMC10730314 38115966

[10] Jensen P , Madsen C, Hauge LJ et al Contact with primary care physicians among adults with pre-existing common mental health problems during the COVID-19 pandemic: a registry-based study from Norway. BMC Health Serv Res 2023;23:1085. 10.1186/s12913-023-10108-3 PMC10568894 37821874

[11] Lovik A , González-Hijón J, Hoffart A et al Elevated symptoms of depression and anxiety among family members and friends of critically ill COVID-19 patients–an observational study of five cohorts across four countries. Lancet Reg Health Eur 2023;33:100733. 10.1016/j.lanepe.2023.100733 PMC10636287 37953992

[12] Lund IO , Jensen P, Madsen C et al Patterns of psychiatric healthcare use during pandemic times among boys and girls with pre-existing diagnoses: a Norwegian nationwide primary and specialist healthcare registry study. BMC Psychiatry 2025;25:68. 10.1186/s12888-024-06422-7 PMC11756222 39844080

[13] Pettersen JH , Hannigan LJ, Gustavson K et al COVID-19 pandemic quarantines and mental health among adolescents in Norway. JAMA Netw Open 2024;7:e2422189–e2422189. 10.1001/jamanetworkopen.2024.22189 PMC11245726 38995642

[14] Pettersen JH , Hegemann L, Gustavson K et al Eating problems among adolescent boys and girls before and during the Covid‐19 pandemic. Int J Eat Disord 2025;58:193–205. 10.1002/eat.24314 PMC11784851 39473346

[15] Sappenfield O et al HRSA (Health Resources and Services Administration) Maternal and Child … 2024.

[16] Woolf SH. The youth mental health crisis in the United States: epidemiology, contributors, and potential solutions. Pediatrics 2025;156:e2025070849. 10.1542/peds.2025-070849 41145167

[17] Danielson ML , Claussen AH, Bitsko RH et al ADHD prevalence among US children and adolescents in 2022: diagnosis, severity, co-occurring disorders, and treatment. J Clin Child Adolesc Psychol 2024;53:343–60. 10.1080/15374416.2024.2335625 PMC11334226 38778436

[18] Gyllenberg D , Marttila M, Sund R et al Temporal changes in the incidence of treated psychiatric and neurodevelopmental disorders during adolescence: an analysis of two national Finnish birth cohorts. Lancet Psychiatry 2018;5:227–36. 10.1016/S2215-0366(18)30038-5 29398636

[19] Minotti C , McKenzie C, Dewandel I et al How does post COVID differ from other post-viral conditions in childhood and adolescence (0–20 years old)? A systematic review. EClinicalMedicine 2024;68:102436. 10.1016/j.eclinm.2024.102436 PMC10850405 38333536

[20] Wang Y , Alcalde-Herraiz M, Güell KL et al Refinement of post-COVID condition core symptoms, subtypes, determinants, and health impacts: a cohort study integrating real-world data and patient-reported outcomes. EBioMedicine 2025;111:105493. 10.1016/j.ebiom.2024.105493 PMC11697707 39662181

[21] Olsen J , Melbye M, Olsen SF et al The Danish National Birth Cohort-its background, structure and aim. Scand J Public Health 2001;29:300–7. 10.1177/14034948010290040201 11775787

[22] Strandberg-Larsen K , Cederkvist L, Aakjær A et al Cohort profile: the Danish National Birth Cohort from foetal life to young adulthood. Int J Epidemiol 2025;54:dyaf083. 10.1093/ije/dyaf083 40527481

[23] Magnus P , Birke C, Vejrup K et al Cohort profile update: the Norwegian mother and child cohort study (MoBa). Int J Epidemiol 2016;45:382–8. 10.1093/ije/dyw029 27063603

[24] Brandlistuen RE , Kristjansson D, Alsaker E et al Cohort Profile Update: The Norwegian Mother, Father and Child Cohort (MoBa). Int J Epidemiol 2025;54:1–9. 10.1093/ije/dyaf139 PMC12367290 40834906

[25] The Danish National Birth Cohort. DNBC Covid-19. 2022. https://www.dnbc.dk/data-available/covid-19

[26] Danielsen S , Joensen A, Andersen PK et al Self-injury, suicidality and eating disorder symptoms in young adults following COVID-19 lockdowns in Denmark. Nat Hum Behav 2023;7:411–9. 10.1038/s41562-022-01511-7 36658210

[27] Groot J , Keller A, Joensen A et al Impact of housing conditions on changes in youth’s mental health following the initial national COVID-19 lockdown: a cohort study. Sci Rep 2022;12:1939. 10.1038/s41598-022-04909-5 PMC8816918 35121742

[28] Joensen A , Danielsen S, Andersen PK et al The impact of the initial and second national COVID-19 lockdowns on mental health in young people with and without pre-existing depressive symptoms. J Psychiatr Res 2022;149:233–42. 10.1016/j.jpsychires.2022.03.001 PMC8902858 35290818

[29] Ebrahimi OV , Bauer DJ, Hoffart A et al A critical period for pandemic adaptation: The evolution of depressive symptomatology in a representative sample of adults across a 17-month period during COVID-19. J Psychopathol Clin Sci 2022;131:881–94. 10.1037/abn0000786 36326629

[30] Ebrahimi OV , Hoffart A, Johnson SU. Physical distancing and mental health during the COVID-19 pandemic: Factors associated with psychological symptoms and adherence to pandemic mitigation strategies. Clin Psychol Sci 2021;9:489–506. 10.1177/2167702621994545

[31] Howe LD , Tilling K, Galobardes B et al Loss to follow-up in cohort studies: bias in estimates of socioeconomic inequalities. Epidemiology 2013;24:1–9. 10.1097/EDE.0b013e31827623b1 PMC5102324 23211345

[32] Nilsen RM , Vollset SE, Gjessing HK et al Self-selection and bias in a large prospective pregnancy cohort in Norway. Paediatr Perinat Epidemiol 2009;23:597–608. 10.1111/j.1365-3016.2009.01062.x 19840297

[33] Rayner C , Hannigan LJ, Badini I et al Quantifying and adjusting for selection biases in the Norwegian Mother, Father and Child Cohort Study using population-wide individual-level registry information. Int J Epidemiol 2026;55:1–11. 10.1093/ije/dyag122 PMC13401474 42501752

[34] Bliddal M , Liew Z, Pottegård A et al Examining nonparticipation in the maternal follow-up within the Danish National Birth Cohort. Am J Epidemiol 2018;187:1511–9. 10.1093/aje/kwy002 PMC6030819 29346474

[35] Jacobsen TN , Nohr EA, Frydenberg M. Selection by socioeconomic factors into the Danish National Birth Cohort. Eur J Epidemiol 2010;25:349–55. 10.1007/s10654-010-9448-2 20349116

[36] Vejrup K , Magnus P, Magnus M. Lost to follow-up in the Norwegian mother, father and child cohort study. Paediatr Perinat Epidemiol 2022;36:300–9. 10.1111/ppe.12821 34797579

[37] Brix N , Ernst A, Lauridsen LLB et al Risk of selection bias due to non‐participation in a cohort study on pubertal timing. Paediatr Perinat Epidemiol 2020;34:668–77. 10.1111/ppe.12679 PMC7754153 32319135

[38] Biele G , Gustavson K, Czajkowski NO et al Bias from self selection and loss to follow-up in prospective cohort studies. Eur J Epidemiol 2019;34:927–38. 10.1007/s10654-019-00550-1 31451995

[39] Madsen KB , Hohwü L, Zhu JL et al Social selection in cohort studies and later representation of childhood psychiatric diagnoses: The Danish National Birth Cohort. Scand J Public Health 2020;48:207–13. 10.1177/1403494817726619 28810815

[40] Frisk NLS , Jørgensen MM, Bæk R et al Characterization of small extracellular vesicles from ovarian cancer patients and pre-diagnostic patient samples: evidence from the Danish blood donor study. PLoS One 2025;20:e0323529. 10.1371/journal.pone.0323529 PMC12080785 40372993

[41] Lovik A , González-Hijón J, Kähler AK et al Mental health indicators in Sweden over a 12-month period during the COVID-19 pandemic–Baseline data of the Omtanke2020 Study. J Affect Disord 2023;322:108–17. 10.1016/j.jad.2022.11.004 PMC9657895 36379324

[42] Ojalo T , Haan E, Kõiv K et al; Estonian Biobank Research Team. Cohort profile update: mental health online survey in the Estonian Biobank (EstBB MHoS). Int J Epidemiol 2024;53:dyae017. 10.1093/ije/dyae017 PMC10881104 38381979

[43] Milani L , Alver M, Laur S et al The Estonian Biobank’s journey from biobanking to personalized medicine. Nat Commun 2025;16:3270. 10.1038/s41467-025-58465-3 PMC11972354 40188112

[44] Magnúsdóttir I , Nygaard AB, Hoffart A et al Acute COVID-19 severity and impaired cognitive function up to 32 months after diagnosis: an observational study. BMC Med 2026;24:311. 10.1186/s12916-026-04856-2 PMC13185386 41965722

[45] Kõiv K , Askeland RB, Christoffersen LAN et al; DBDS Genetic Consortium. Psychiatric genetic liability is associated with the severity of COVID-19 and other acute respiratory infections: an observational study across five Northern European countries. Genome Med 2026, online ahead of print, 10.1186/s13073-026-01723-w PMC13692891 42919319

[46] Quinn L , Caspersen IH, Magnúsdóttir I, et al; DBDS Genetic Consortium. The role of genetic liability for psychiatric disorders and personality traits in post covid syndrome: data from three Nordic population cohorts. EClinicalMedicine 2026;95:103928. 10.1016/j.eclinm.2026.103928 PMC13185856 42163972

[47] Chourpiliadis C , Lovik A, Kähler AK et al Short-term improvement of mental health after a COVID-19 vaccination. PLoS One 2023;18:e0280587. 10.1371/journal.pone.0280587 PMC9931115 36791070

