# Supplementary material for: Cohort Profile Update: COVIDMENT: COVID-19 cohorts on mental health across six nations
Source: Int J Epidemiol. 2026 Oct 26;55(6):dyag233. doi: 10.1093/ije/dyag233 (PMC13706617; doi:10.1093/ije/dyag233)
Supplement: Supplementary file 1 [file dyag233_supplementary_data.docx]

**Supplementary file for Cohort Profile Update: COVIDMENT: COVID-19 cohorts on mental health across six nations**

Helga Ask, Johanne Hagen Pettersen, Mary M Barker, Lea Arregui Nordahl Christoffersen, Sverre Urnes Johnson, Kadri Kõiv, Ingibjörg Magnusdottir, Hannah Milbourn, Anders B Nygaard, Ida Scheel Rasmussen, Thor Aspelund, Sonja Hellegjerde Brunvoll, Archie Campbell, Ida Henriette Caspersen, John Arne Dahl, Christian Erikstrup, Stephanie Le Hellard, Anikó Lovik, Arna Hauksdóttir, Hilde Heiro, Asle Hoffart, Bitten Aagaard Jensen, Anna K Kähler, Karl Trygve Kalleberg, Shiyu Li, Christian Madsen, Gunhild Alvik Nyborg, Sisse Rye Ostrowski, Rachel Elizabeth Ramsey, Mägi Reedik, Erik Sørensen, Edda Bjork Thordardottir, Mie Topholm-Bruun, Nicoline Uglebjerg, Tian Xiao, Arne Søraas, Kelli Lehto, Fang Fang, Ole Birger Pedersen, Ole A. Andreassen, Omid V. Ebrahimi, Ingunn Olea Lund, Katrine Strandberg-Larsen, Ragnhild Brandlistuen, Unnur A Valdimarsdóttir

Contents

[1.	Supplementary Table1: COVIDMENT COHORTS	2](#_Toc237952894)

[2.	Supplementary methods: Measures	2](#_Toc237952895)

[Outcomes MoBa	3](#_Toc237952896)

[Covariates MoBa	4](#_Toc237952897)

[Outcomes DNBC	4](#_Toc237952898)

[Covariates DNBC	6](#_Toc237952899)

[3.	Supplementary methods: Analyses	6](#_Toc237952900)

[4.	Supplementary results DID analyses MoBa:	7](#_Toc237952901)

[Short mood and feeling questionnaire (SMFQ)	7](#_Toc237952902)

[Screen for Child Anxiety Related Emotional Disorders (SCARED)	9](#_Toc237952903)

[ADHD traits	12](#_Toc237952904)

[Hours of sleep	13](#_Toc237952905)

[Hours of physical activity	15](#_Toc237952906)

[Number of close friends	17](#_Toc237952907)

[Overview of results (MoBa)	19](#_Toc237952908)

[5.	Supplementary results DID analyses DNBC:	20](#_Toc237952909)

[Number of close friends	20](#_Toc237952910)

[Self-rated health	20](#_Toc237952911)

[Quality of life	21](#_Toc237952912)

[Sleep	21](#_Toc237952913)

[Prosocial behavior	22](#_Toc237952914)

[Internalizing traits	22](#_Toc237952915)

[Externalizing traits	23](#_Toc237952916)

[6.	References	24](#_Toc237952917)

[Author contributions	25](#_Toc237952918)

## 1. Supplementary Table1: COVIDMENT COHORTS

|  | | **N initial** | **N last** | **Date last data collection** |
| --- | --- | --- | --- | --- |
| **ORIGINAL COVIDMENT COHORTS** | |  |  |  |
| Norway | MoBa (adults Covid data collections) | 132 486* | 34 062 | November, 2025 |
| Iceland | Resilience cohort | 22 849* | 11 564 | September, 2022 - December, 2022 (pending, 2026) |
| Denmark | DBDS** | 71 562* | 30 297 | July, 2022 |
| Sweden | Omtanke2020 | 28 614* | 15 239 | December, 2024 - March, 2025 |
| Norway | BryDeg | 19 343* | 3 663 | December, 2023 |
| Scotland | Generation Scotland CovidLIfe study | 18 518* | 10 386 | February, 2021 |
| Estonia | EstBB-C19 | 13 329* | 14 451 | May, 2020 - August, 2021 |
| Estonia | EstBB MHoS | 86 116* | 86 244 | March - July, 2021  (April – August 2026****) |
| **TOTAL N:** |  | **392 817*** | **204 519** |  |
| **NEW COVIDMENT COHORTS** | |  |  |  |
| Norway | MAP-19 | 10 061 | 2 870 | March, 2023 |
| Denmark | DNBC | 53 323 | 1 531*** | April - May, 2021 |
| Norway | NCC | 182 560 | 90 751 | October, 2023 - March, 2026 |
| Scotland | TeenCovidLife | 7 058 | 7 058 | May, 2021 |
| Norway | MoBa – 14- yrs + covid 16 years | 33 042 | 33 042 | December, 2024 (14-year) |
| **TOTAL:** |  | **283 136** | **135 252** |  |
| **N TOTAL RECENT DATA COLLECTION** | |  | **339 771** |  |

Supplementary Table 1. Number of participants (N) from the original and new COVIDMENT cohorts. **N initial** refers to the number of participants in the first data collection conducted early in the pandemic; **N last** refers to the number of participants in the most recent data collection, with the timing of data collection varying across cohorts. The difference between these values should not be interpreted as a measure of participant retention, as some cohorts continued to recruit or invite new participants during follow-up, resulting in a larger number of participants at later assessments than at baseline. * = Initial numbers from the COVIDMENT Cohort profile published in 2022. **The DBDS source population changed between survey rounds: the initial surveys included donation-active participants and retired donors, whereas the latest surveys included only DBDS participants. Also, initial N for DBDS reflects total N across several data collections; ***Refers to N participants in the DNBC Wave 7 of the covid data collections, only recruiting participants responding to all previous (1-6) waves of DBNC covid data collection; ****A new EstBB MHoS data collection is completed with around 60 000 respondents, final participation numbers pending

## 2. Supplementary methods: Measures

### Outcomes MoBa

Mothers responded to questionnaires when their children were both age 8 and 14 with similar variables measured. The 14-year questionnaire was responded to between 2017 and 2024. For all multi-item outcome measures, a mean score was calculated for participants who responded to at least 50% of the items. The resulting mean scores were then standardized using the *scale()* function in R.

**Depression symptoms**

Depression symptoms were measured using items from the Short Mood and Feelings Questionnaire (SMFQ)^1^. The 13-item short version (SFMQ) of the original 32-item Mood and Feelings Questionnaire (MFQ)^2^ was included in the 8-year questionnaire, while six selected items from SMFQ were also included in the 14-year questionnaire. The six overlapping items were used in this study and scored from 0 (Not true) to 3 (True). In addition to the parent-report in both questionnaires, self-reported depression symptoms were also assessed in the 14-year questionnaire with the 13-item short version (SMFQ). The six items were also selected here and scored similarly as the parent-reported items.

**Anxiety symptoms**

Anxiety symptoms were assessed using the 5-item short version of the Screen for Child Anxiety Related Emotional Disorders (SCARED)^3,4^. The items were scored from 0 (Not true) to 3 (True/Very true). In addition to parent-reports at 8 and 14 years, anxiety symptoms were also assessed with similar items (although slightly different wording) in a self-report questionnaire at 14 years.

**ADHD traits**

ADHD traits were measured using the 18 ADHD items from the Rating Scale for Disruptive Behavior Disorders (RS-DBD)^5^. The items were scored from 0 (Never/rarely) to 3 (Very often).

**Sleep**

Sleep duration was assessed using a single item asking: “*About how many hours does the child usually sleep on weeknights?*”. Response options differed slightly between the questionnaire waves. In the 8-year questionnaire, response categories were: *“8 hours or less”, ”9 hours”, ”10 hours”, ”11 hours”, and ”12 hours or more”*. These response categories were coded 0-4. In the 14-year questionnaire, response categories were: *“6 hours or less”, “7 hours”, “8 hours”, “9 hours”, “10 hours”, and “11 hours or more”.* These response categories were coded 0-4 and 0-5.

To compare sleep across questionnaire waves, both sleep variables were rescaled to 0-1 by dividing the original score by the maximum value within each questionnaire. This allows both sleep variables to be on the same scale, with a value of 0 representing the shortest sleep category and a value of 1 representing the longest sleep category within each questionnaire.

**Close friends**

The number of close friends was assessed using a single item asking: “*Approximately how many close friends does your child have (not including siblings)?”*. Response options in both questionnaires were: *“None”, “1 friend”, “2-3 friends” or “4+ friends”* and the variable was scored from 0-3.

**Physical activity**

Hours of physical activity was measured using one item asking: *“Outside of school hours: Approximately how many hours per week is your child physically active/takes part in sports (football, handball, skiing, gymnastics/dance, etc.)?* Hours of physical activity in after-school programs (SFO) were included*.* The response options in both questionnaires were: *“Less than 1 hour”, “1-2 hours”, “3-4 hours”, “5-7 hours”, “8-10 hours”,* and *“11 hours or more”* and coded from 0-5. In the 8-year questionnaire, physical activity was assessed separately for summer and winter. The two items were first coded identically and then combined by calculating the average of the summer and winter scores, yielding a single physical activity measure for age 8 to be comparable to the single item from the 14-year questionnaire. In addition to parent reported physical activity, adolescents were also asked to report on their own physical activity in the 14-year questionnaire with a similar item and response options.

### Covariates MoBa

**Pre-pandemic and pandemic groups**

Participants were divided into pre-pandemic and pandemic groups based on the timing of the response for the 14-year questionnaire. Those responding prior to March 1^st^, 2020, were grouped as “Pre-pandemic” while those responding after the index date were grouped as “Pandemic”. This threshold was selected as the mothers were asked to respond based on their child’s behavior during the last two weeks for several of the questionnaire scales.

**Parental education**

Mothers and fathers’ education in 2020 (latest date available at this time) was retrieved from Statistics Norway (SSB). Education was coded as follows: 0 (none/compulsory), 1 (upper secondary), 2 (bachelor’s degree) and 3 (master’s or PhD) for mothers and fathers separately. A variable for parental education was created by selecting the highest education between mothers and fathers.

Child age at the 14-year questionnaire (14-16 years), maternal age at birth (17-46 years) and child sex (0 - male, 1 - female) were also included. Among mothers participating in MoBa with several children, the first-born child was selected.

### Outcomes DNBC

Children and adolescents responded to questionnaires when they were age 11 and 18 years. The 18-year questionnaire was responded to between 2014 and 2021. For all multi-item outcome measures, a mean score was calculated for participants who responded to at least 50% of the items. The resulting mean scores were then standardized using the *scale()* function in R.

**Emotional problems**

The Strengths and Difficulties Questionnaire^6-8^ was used to assess emotional problems, using the broader internalising scale, which is recommended for non-at-risk groups. The internalising subscale is designed to assess the internally directed emotional difficulties by combining the emotional subscale and the subscale concerning peer problems into one scale. The internalising scale includes ten items, each scored on a 3-point Likert scale with the response options “*Not true*”, “*Somewhat true*” and “*Certainly true*” producing a total score ranging between 0 and 20, where higher scores reflect more internalising difficulties.

**Behavioural problems**

The Strengths and Difficulties Questionnaire was used to assess behavioural problems, applying the broader externalising scale, which is recommended for non-at-risk groups. The externalising subscale is designed to assess the outward-directed behavioural difficulties by combining the hyperactivity/inattention subscale and the conduct problems subscale into one scale. Thus, the externalising scale includes ten items, each scored on a 3-point Likert scale with the response options “*Not true*”, “*Somewhat true*” and “*Certainly true*”, producing a total score ranging between 0 and 20, where higher scores reflect more difficulties.

**Social skills**

Social skills were based on the Prosocial Scale, one of the five subscales in the Strengths and Difficulties Questionnaire. The subscale focuses on the participants’ positive social behaviours towards others. It consists of five items, each scored on a 3-point Likert scale with the response options “*Not true*”, “*Somewhat true*” and “*Certainly true*” producing a total score between 0 and 10. The purpose of the questions is to capture the respondents’ social strengths, such as empathy and helpfulness. Higher scores reflect stronger prosocial skills.

**Subjective health**

The participants’ overall health was assessed using two similar questions at the 11‑ and the 18‑year follow-up. At age 11, the children were asked: “*How would you rate your own health?*” with the response categories “*Excellent*”, “*Good*”, “*Not so good*” and “*Poor*”. At the age of 18, the adolescents were asked: “*How would you describe your overall health?*” with the response categories “*Outstanding*”, “*Very good*”, “*Good*”, “*Less good*” and “*Bad*”. These subjective health questions were based on and adapted from the 12 Item Short Form Health Survey (SF‑12)^9^. A numeric score was created for both age groups and higher scores indicate poorer health.

**Quality of life**

Quality of life was assessed using the Cantril Self-Anchoring Striving Scale^10^ (i.e. the Cantril ladder) as a measure of overall life satisfaction and subjective well-being. The participants were asked to imagine a ladder with steps numbered from 0 to 10, where 0 represents “*the worst life ever*” and 10 represents “*the best life ever*”, thus higher scores indicate better perceived quality of life. They were then guided to place themselves on the ladder based on their current situation.

**Sleep**

Sleep was based on their reported hours of sleep. The participants’ sleep duration was calculated based on the questions asking: “*At what time do you usually go to sleep on weekdays?*” and “*When do you get woken or wake up on weekdays*?”. Similar questions were asked for the weekend: “*At what time do you usually go to sleep during the weekend?*” and “*When do you get woken or wake up during the weekend?*”. Based on their answers, a weighted sleep duration that took both weekday and weekend sleep into account was calculated.

**Friends**

In the DNBC, ‘friends’ was based on the number of friends reported by the participants themselves. At age 11, the children were asked: “*How many of your close friends are boys at the moment?”* and “*How many of your close friends are girls at the moment?*” These two answers were combined to create the total number of close friends at age 11. At age 18, the adolescents were asked: “*How many close friends do you have?*”.

### Covariates DNBC

**Pre-pandemic and pandemic groups**

Participants were divided into pre-pandemic and pandemic groups based on the timing of the response for the 18-year questionnaire. Those responding prior to March 1^st^, 2020, were grouped as “Pre-pandemic” whereas those responding after were grouped as “Pandemic”.

**Covariates**

Parental education was retrieved from Statistics Denmark. Parental education was defined as the highest attained education among the participants’ parents when the participants turned 18 years, thus selecting the educational level that was highest among the mother and the father. The classification of parental education was based on the ISCED levels and inspired by SEPLINE: Socioeconomic Position in Epidemiological Research - A National Guideline on Danish Registry Data guideline^11^: basic (e.g. primary and lower secondary school), short (e.g. vocational education and training), medium (e.g. bachelor’s programs) and high (e.g. master’s programs or above).

Participants’ age when responding to the 18-years follow-up, the maternal age when the participants turned 18 and the participants’ sex were also included. Among mothers with several children, the first-born child was selected.

## 3. Supplementary methods: Analyses

For MoBa, two sets of sensitivity analyses were also conducted

**Sensitivity 1: Including individuals responding before 2022**

**Sensitivity 2: Including self-report at Q14 (instead of mother-report)**

## 4. Supplementary results DID analyses MoBa:

### Short mood and feeling questionnaire (SMFQ)

**Main results**

| **SMFQ - standardized** | **Unadjusted model** | | | **Adjusted model** | | |
| --- | --- | --- | --- | --- | --- | --- |
|  | **β** | **95% CI** | **P-value** | **β** | **95% CI** | **P-value** |
| **Girls** |  |  |  |  |  |  |
| Time Q14 (ref = Q8) | 0.167 | [0.128, 0.205] | **<0.001** | 0.167 | [0.128, 0.205] | **<0.001** |
| Pandemic group (ref = pre-pandemic group) | 0.040 | [0.010, 0.070] | **0.009** | 0.046 | [0.016, 0.077] | **0.003** |
| Pandemic group x time (DID) | 0.104 | [0.055, 0.154] | **<0.001** | 0.104 | [0.054, 0.153] | **<0.001** |
| Adjusted R^2^ |  |  | 0.016 |  |  | 0.017 |
| Observations |  |  | 19,546 |  |  | 19,530 |
|  |  |  |  |  |  |  |
| **Boys** |  |  |  |  |  |  |
| Time Q14 (ref = Q8) | -0.067 | [-0.106, -0.027] | **<0.001** | -0.066 | [-0.106, -0.027] | **0.001** |
| Pandemic group (ref = pre-pandemic group) | 0.089 | [0.052, 0.125] | **<0.001** | 0.099 | [0.062, 0.135] | **<0.001** |
| Pandemic group x time (DID) | -0.027 | [-0.076, 0.023] | 0.290 | -0.027 | [-0.077, 0.023] | 0.287 |
| Adjusted R^2^ |  |  | 0.003 |  |  | 0.005 |
| Observations |  |  | 19,528 |  |  | 19,512 |

**Note**: Cluster-robust standard errors used to compute 95% confidence intervals. Adjusted model controls for maternal and child covariates (age child at Q14, maternal age, education)

**Sensitivity 1: Including individuals responding before 2022**

| **SMFQ - standardized** | **Unadjusted model** | | | **Adjusted model** | | |
| --- | --- | --- | --- | --- | --- | --- |
|  | **β** | **95% CI** | **P-value** | **β** | **95% CI** | **P-value** |
| **Girls** |  |  |  |  |  |  |
| Time Q14 (ref = Q8) | 0.171 | [0.132, 0.210] | **<0.001** | 0.171 | [0.132, 0.210] | **<0.001** |
| Pandemic group (ref = pre-pandemic group) | 0.019 | [-0.015, 0.052] | 0.274 | 0.025 | [-0.008, 0.058] | 0.142 |
| Pandemic group x time (DID) | 0.077 | [0.022, 0.131] | **0.006** | 0.076 | [0.022, 0.131] | **0.006** |
| Adjusted R^2^ |  |  | 0.012 |  |  | 0.014 |
| Observations |  |  | 15,494 |  |  | 15,484 |
|  |  |  |  |  |  |  |
| **Boys** |  |  |  |  |  |  |
| Time Q14 (ref = Q8) | -0.069 | [-0.109, -0.028] | **<0.001** | -0.068 | [-0.109, -0.027] | **0.001** |
| Pandemic group (ref = pre-pandemic group) | 0.062 | [0.022, 0.102] | **0.002** | 0.071 | [0.031, 0.111] | **<0.001** |
| Pandemic group x time (DID) | -0.050 | [-0.104, 0.004] | 0.071 | -0.050 | [-0.105, 0.004] | 0.070 |
| Adjusted R^2^ |  |  | 0.003 |  |  | 0.005 |
| Observations |  |  | 15,516 |  |  | 15,504 |

**Note**: Cluster-robust standard errors used to compute 95% confidence intervals. Adjusted model controls for maternal and child covariates (age child at Q14, maternal age, education)

**Sensitivity 2: Including self-report at Q14 (instead of mother-report)**

| **SMFQ - standardized** | **Unadjusted model** | | | **Adjusted model** | | |
| --- | --- | --- | --- | --- | --- | --- |
|  | **β** | **95% CI** | **P-value** | **β** | **95% CI** | **P-value** |
| **Girls** |  |  |  |  |  |  |
| Time Q14 (ref = Q8) | 1.056 | [1.017, 1.095] | **<0.001** | 1.056 | [1.017, 1.095] | **<0.001** |
| Pandemic group (ref = pre-pandemic group) | 0.016 | [-0.003, 0.036] | 0.091 | 0.019 | [-0.000, 0.039] | 0.050 |
| Pandemic group x time (DID) | 0.130 | [0.079, 0.181] | **<0.001** | 0.128 | [0.077, 0.179] | **<0.001** |
| Adjusted R^2^ |  |  | 0.323 |  |  | 0.323 |
| Observations |  |  | 15,258 |  |  | 15,250 |
|  |  |  |  |  |  |  |
| **Boys** |  |  |  |  |  |  |
| Time Q14 (ref = Q8) | 0.617 | [0.572, 0.662] | **<0.001** | 0.617 | [0.572, 0.662] | **<0.001** |
| Pandemic group (ref = pre-pandemic group) | 0.051 | [0.022, 0.080] | **<0.001** | 0.053 | [0.023, 0.082] | **<0.001** |
| Pandemic group x time (DID) | 0.071 | [0.012, 0.130] | **0.019** | 0.072 | [0.013, 0.131] | **0.017** |
| Adjusted R^2^ |  |  | 0.111 |  |  | 0.111 |
| Observations |  |  | 14,372 |  |  | 14,362 |

**Note**: Cluster-robust standard errors used to compute 95% confidence intervals. Adjusted model controls for maternal and child covariates (age child at Q14, maternal age, education)

### Screen for Child Anxiety Related Emotional Disorders (SCARED)

**Main results**

| **SCARED - standardized** | **Unadjusted model** | | | **Adjusted model** | | |
| --- | --- | --- | --- | --- | --- | --- |
|  | **β** | **95% CI** | **P-value** | **β** | **95% CI** | **P-value** |
| **Girls** |  |  |  |  |  |  |
| Time Q14 (ref = Q8) | -0.242 | [-0.277, -0.207] | **<0.001** | -0.242 | [-0.277, -0.206] | **<0.001** |
| Pandemic group (ref = pre-pandemic group) | 0.076 | [0.035, 0.116] | **<0.001** | 0.082 | [0.041, 0.122] | **<0.001** |
| Pandemic group x time (DID) | 0.042 | [-0.004, 0.088] | 0.076 | 0.042 | [-0.005, 0.088] | 0.079 |
| Adjusted R^2^ |  |  | 0.014 |  |  | 0.015 |
| Observations |  |  | 19,616 |  |  | 19,600 |
|  |  |  |  |  |  |  |
| **Boys** |  |  |  |  |  |  |
| Time Q14 (ref = Q8) | -0.416 | [-0.453, -0.378] | **<0.001** | -0.415 | [-0.453, -0.378] | **<0.001** |
| Pandemic group (ref = pre-pandemic group) | 0.055 | [0.010, 0.100] | **0.016** | 0.056 | [0.011, 0.102] | **0.015** |
| Pandemic group x time (DID) | -0.002 | [-0.049, 0.045] | 0.939 | -0.002 | [-0.050, 0.045] | 0.920 |
| Adjusted R^2^ |  |  | 0.044 |  |  | 0.044 |
| Observations |  |  | 19,588 |  |  | 19,572 |

**Note**: Cluster-robust standard errors used to compute 95% confidence intervals. Adjusted model controls for maternal and child covariates (age child at Q14, maternal age, education)

**Sensitivity 1: Including individuals responding before 2022**

| **SCARED - standardized** | **Unadjusted model** | | | **Adjusted model** | | |
| --- | --- | --- | --- | --- | --- | --- |
|  | **β** | **95% CI** | **P-value** | **β** | **95% CI** | **P-value** |
| **Girls** |  |  |  |  |  |  |
| Time Q14 (ref = Q8) | -0.247 | [-0.283, -0.211] | **<0.001** | -0.247 | [-0.283, -0.211] | **<0.001** |
| Pandemic group (ref = pre-pandemic group) | 0.044 | [-0.001, 0.089] | 0.054 | 0.051 | [0.006, 0.096] | **0.028** |
| Pandemic group x time (DID) | 0.051 | [-0.000, 0.103] | 0.052 | 0.051 | [-0.001, 0.102] | 0.055 |
| Adjusted R^2^ |  |  | 0.013 |  |  | 0.041 |
| Observations |  |  | 15,554 |  |  | 15,544 |
|  |  |  |  |  |  |  |
| **Boys** |  |  |  |  |  |  |
| Time Q14 (ref = Q8) | -0.424 | [-0.462, -0.385] | **<0.001** | -0.423 | [-0.462, -0.385] | **<0.001** |
| Pandemic group (ref = pre-pandemic group) | 0.036 | [-0.014, 0.085] | 0.158 | 0.040 | [-0.010, 0.090] | 0.118 |
| Pandemic group x time (DID) | -0.012 | [-0.063, 0.040] | 0.656 | -0.011 | [-0.063, 0.040] | 0.673 |
| Adjusted R^2^ |  |  | 0.046 |  |  | 0.047 |
| Observations |  |  | 15,552 |  |  | 15,540 |

**Note**: Cluster-robust standard errors used to compute 95% confidence intervals. Adjusted model controls for maternal and child covariates (age child at Q14, maternal age, education)

**Sensitivity 2: Including self-report at Q14 (instead of mother-report)**

| **SCARED - standardized** | **Unadjusted model** | | | **Adjusted model** | | |
| --- | --- | --- | --- | --- | --- | --- |
|  | **β** | **95% CI** | **P-value** | **β** | **95% CI** | **P-value** |
| **Girls** |  |  |  |  |  |  |
| Time Q14 (ref = Q8) | 0.712 | [0.669, 0.755] | **<0.001** | 0.712 | [0.669, 0.755] | **<0.001** |
| Pandemic group (ref = pre-pandemic group) | 0.040 | [0.010, 0.069] | **0.009** | 0.045 | [0.015, 0.074] | **0.003** |
| Pandemic group x time (DID) | 0.010 | [-0.047, 0.067] | 0.741 | 0.008 | [-0.049, 0.065] | 0.794 |
| Adjusted R^2^ |  |  | 0.129 |  |  | 0.130 |
| Observations |  |  | 15,244 |  |  | 15,236 |
|  |  |  |  |  |  |  |
| **Boys** |  |  |  |  |  |  |
| Time Q14 (ref = Q8) | 0.071 | [0.023, 0.118] | **0.003** | 0.071 | [0.024, 0.118] | **0.003** |
| Pandemic group (ref = pre-pandemic group) | 0.045 | [0.002, 0.088] | **0.043** | 0.045 | [0.002, 0.089] | **0.041** |
| Pandemic group x time (DID) | -0.026 | [-0.088, 0.037] | 0.418 | -0.025 | [-0.088, 0.037] | 0.427 |
| Adjusted R^2^ |  |  | 0.001 |  |  | 0.001 |
| Observations |  |  | 14,252 |  |  | 14,244 |

**Note**: Cluster-robust standard errors used to compute 95% confidence intervals. Adjusted model controls for maternal and child covariates (age child at Q14, maternal age, education)

### ADHD traits

**Main results**

| **ADHD - standardized** | **Unadjusted model** | | | **Adjusted model** | | |
| --- | --- | --- | --- | --- | --- | --- |
|  | **β** | **95% CI** | **P-value** | **β** | **95% CI** | **P-value** |
| **Girls** |  |  |  |  |  |  |
| Time Q14 (ref = Q8) | -0.492 | [-0.522, -0.463] | **<0.001** | -0.492 | [-0.522, -0.463] | **<0.001** |
| Pandemic group (ref = pre-pandemic group) | 0.073 | [0.032, 0.115] | **<0.001** | 0.087 | [0.046, 0.129] | **<0.001** |
| Pandemic group x time (DID) | 0.156 | [0.118, 0.195] | **<0.001** | 0.156 | [0.118, 0.195] | **<0.001** |
| Adjusted R^2^ |  |  | 0.045 |  |  | 0.049 |
| Observations |  |  | 19,604 |  |  | 19,588 |
|  |  |  |  |  |  |  |
| **Boys** |  |  |  |  |  |  |
| Time Q14 (ref = Q8) | -0.446 | [-0.476, -0.416] | **<0.001** | -0.446 | [-0.476, -0.416] | **<0.001** |
| Pandemic group (ref = pre-pandemic group) | 0.047 | [0.005, 0.089] | **0.027** | 0.065 | [0.023, 0.107] | **0.002** |
| Pandemic group x time (DID) | 0.096 | [0.058, 0.133] | **<0.001** | 0.096 | [0.058, 0.133] | **<0.001** |
| Adjusted R^2^ |  |  | 0.039 |  |  | 0.043 |
| Observations |  |  | 19,560 |  |  | 19,544 |

**Note**: Cluster-robust standard errors used to compute 95% confidence intervals. Adjusted model controls for maternal and child covariates (age child at Q14, maternal age, education)

**Sensitivity 1: Including individuals responding before 2022**

| **ADHD - standardized** | **Unadjusted model** | | | **Adjusted model** | | |
| --- | --- | --- | --- | --- | --- | --- |
|  | **β** | **95% CI** | **P-value** | **β** | **95% CI** | **P-value** |
| **Girls** |  |  |  |  |  |  |
| Time Q14 (ref = Q8) | -0.506 | [-0.537, -0.476] | **<0.001** | -0.506 | [-0.537, -0.476] | **<0.001** |
| Pandemic group (ref = pre-pandemic group) | 0.065 | [0.019, 0.110] | **0.005** | 0.076 | [0.030, 0.121] | **0.001** |
| Pandemic group x time (DID) | 0.117 | [0.074, 0.160] | **<0.001** | 0.116 | [0.073, 0.160] | **<0.001** |
| Adjusted R^2^ |  |  | 0.053 |  |  | 0.057 |
| Observations |  |  | 15,538 |  |  | 15,528 |
|  |  |  |  |  |  |  |
| **Boys** |  |  |  |  |  |  |
| Time Q14 (ref = Q8) | -0.454 | [-0.484, -0.423] | **<0.001** | -0.454 | [-0.484, -0.423] | **<0.001** |
| Pandemic group (ref = pre-pandemic group) | 0.028 | [-0.018, 0.073] | 0.233 | 0.045 | [-0.001, 0.090] | 0.055 |
| Pandemic group x time (DID) | 0.053 | [0.012, 0.093] | **0.011** | 0.053 | [0.012, 0.093] | **0.010** |
| Adjusted R^2^ |  |  | 0.046 |  |  | 0.051 |
| Observations |  |  | 15,536 |  |  | 15,524 |

**Note**: Cluster-robust standard errors used to compute 95% confidence intervals. Adjusted model controls for maternal and child covariates (age child at Q14, maternal age, education)

### Hours of sleep

**Main results**

| **Sleep - standardized** | **Unadjusted model** | | | **Adjusted model** | | |
| --- | --- | --- | --- | --- | --- | --- |
|  | **β** | **95% CI** | **P-value** | **β** | **95% CI** | **P-value** |
| **Girls** |  |  |  |  |  |  |
| Time Q14 (ref = Q8) | -0.533 | [-0.587, -0.479] | **<0.001** | -0.533 | [-0.587, -0.479] | **<0.001** |
| Pandemic group (ref = pre-pandemic group) | 0.019 | [-0.033, 0.071] | 0.471 | 0.017 | [-0.035, 0.069] | 0.522 |
| Pandemic group x time (DID) | -0.162 | [-0.223, -0.100] | **<0.001** | -0.163 | [-0.225, -0.101] | **<0.001** |
| Adjusted R^2^ |  |  | 0.110 |  |  | 0.113 |
| Observations |  |  | 16,162 |  |  | 16,146 |
|  |  |  |  |  |  |  |
| **Boys** |  |  |  |  |  |  |
| Time Q14 (ref = Q8) | -0.454 | [-0.513, -0.396] | **<0.001** | -0.454 | [-0.513, -0.396] | **<0.001** |
| Pandemic group (ref = pre-pandemic group) | 0.013 | [-0.042, 0.068] | 0.637 | 0.011 | [-0.044, 0.067] | 0.683 |
| Pandemic group x time (DID) | -0.086 | [-0.152, -0.021] | **0.010** | -0.086 | [-0.152, -0.021] | **0.010** |
| Adjusted R^2^ |  |  | 0.069 |  |  | 0.072 |
| Observations |  |  | 16,180 |  |  | 16,166 |

**Note**: Cluster-robust standard errors used to compute 95% confidence intervals. Adjusted model controls for maternal and child covariates (age child at Q14, maternal age, education)

**Sensitivity 1: Including individuals responding before 2022**

| **Sleep - standardized** | **Unadjusted model** | | | **Adjusted model** | | |
| --- | --- | --- | --- | --- | --- | --- |
|  | **β** | **95% CI** | **P-value** | **β** | **95% CI** | **P-value** |
| **Girls** |  |  |  |  |  |  |
| Time Q14 (ref = Q8) | -0.537 | [-0.592, -0.482] | **<0.001** | -0.537 | [-0.592, -0.482] | **<0.001** |
| Pandemic group (ref = pre-pandemic group) | 0.006 | [-0.049, 0.060] | 0.841 | 0.006 | [-0.049, 0.060] | 0.837 |
| Pandemic group x time (DID) | -0.149 | [-0.214, -0.084] | **<0.001** | -0.150 | [-0.215, -0.085] | **<0.001** |
| Adjusted R^2^ |  |  | 0.105 |  |  | 0.108 |
| Observations |  |  | 12,164 |  |  | 12,154 |
|  |  |  |  |  |  |  |
| **Boys** |  |  |  |  |  |  |
| Time Q14 (ref = Q8) | -0.460 | [-0.519, -0.401] | **<0.001** | -0.460 | [-0.519, -0.401] | **<0.001** |
| Pandemic group (ref = pre-pandemic group) | 0.002 | [-0.056, 0.060] | 0.937 | 0.002 | [-0.056, 0.060] | 0.940 |
| Pandemic group x time (DID) | -0.087 | [-0.156, -0.017] | **0.014** | -0.087 | [-0.156, -0.018] | **0.014** |
| Adjusted R^2^ |  |  | 0.069 |  |  | 0.072 |
| Observations |  |  | 12,204 |  |  | 12,194 |

**Note**: Cluster-robust standard errors used to compute 95% confidence intervals. Adjusted model controls for maternal and child covariates (age child at Q14, maternal age, education)

### Hours of physical activity

**Main results**

| **Physical activity - standardized** | **Unadjusted model** | | | **Adjusted model** | | |
| --- | --- | --- | --- | --- | --- | --- |
|  | **β** | **95% CI** | **P-value** | **β** | **95% CI** | **P-value** |
| **Girls** |  |  |  |  |  |  |
| Time Q14 (ref = Q8) | -0.201 | [-0.243, -0.159] | **<0.001** | -0.201 | [-0.243, -0.159] | **<0.001** |
| Pandemic group (ref = pre-pandemic group) | -0.007 | [-0.044, 0.030] | 0.715 | -0.023 | [-0.059, 0.014] | 0.231 |
| Pandemic group x time (DID) | -0.082 | [-0.135, -0.028] | **0.003** | -0.081 | [-0.135, -0.028] | **0.003** |
| Adjusted R^2^ |  |  | 0.017 |  |  | 0.021 |
| Observations |  |  | 19,376 |  |  | 19,360 |
|  |  |  |  |  |  |  |
| **Boys** |  |  |  |  |  |  |
| Time Q14 (ref = Q8) | -0.355 | [-0.396, -0.314] | **<0.001** | -0.356 | [-0.397, -0.315] | **<0.001** |
| Pandemic group (ref = pre-pandemic group) | 0.001 | [-0.037, 0.038] | 0.976 | -0.021 | [-0.058, 0.016] | 0.269 |
| Pandemic group x time (DID) | 0.010 | [-0.040, 0.061] | 0.686 | 0.011 | [-0.040, 0.062] | 0.672 |
| Adjusted R^2^ |  |  | 0.030 |  |  | 0.036 |
| Observations |  |  | 19,382 |  |  | 19,366 |
|  |  |  |  |  |  |  |

**Note**: Cluster-robust standard errors used to compute 95% confidence intervals. Adjusted model controls for maternal and child covariates (age child at Q14, maternal age, education)

**Sensitivity 1: Including individuals responding before 2022**

| **Physical activity - standardized** | **Unadjusted model** | | | **Adjusted model** | | |
| --- | --- | --- | --- | --- | --- | --- |
|  | **β** | **95% CI** | **P-value** | **β** | **95% CI** | **P-value** |
| **Girls** |  |  |  |  |  |  |
| Time Q14 (ref = Q8) | -0.202 | [-0.244, -0.159] | **<0.001** | -0.202 | [-0.244, -0.159] | **<0.001** |
| Pandemic group (ref = pre-pandemic group) | -0.012 | [-0.052, 0.027] | 0.539 | -0.026 | [-0.066, 0.013] | 0.195 |
| Pandemic group x time (DID) | -0.079 | [-0.137, -0.021] | **0.007** | -0.079 | [-0.137, -0.021] | **0.008** |
| Adjusted R^2^ |  |  | 0.016 |  |  | 0.021 |
| Observations |  |  | 15,374 |  |  | 15,364 |
|  |  |  |  |  |  |  |
| **Boys** |  |  |  |  |  |  |
| Time Q14 (ref = Q8) | -0.355 | [-0.395, -0.314] | **<0.001** | -0.355 | [-0.396, -0.314] | **<0.001** |
| Pandemic group (ref = pre-pandemic group) | -0.002 | [-0.042, 0.038] | 0.918 | -0.021 | [-0.061, 0.019] | 0.305 |
| Pandemic group x time (DID) | -0.018 | [-0.072, 0.037] | 0.525 | -0.017 | [-0.072, 0.038] | 0.541 |
| Adjusted R^2^ |  |  | 0.033 |  |  | 0.039 |
| Observations |  |  | 15,386 |  |  | 15,374 |
|  |  |  |  |  |  |  |

**Note**: Cluster-robust standard errors used to compute 95% confidence intervals. Adjusted model controls for maternal and child covariates (age child at Q14, maternal age, education)

**Sensitivity 2: Including self-report at Q14 (instead of mother-report)**

| **Physical activity - standardized** | **Unadjusted model** | | | **Adjusted model** | | |
| --- | --- | --- | --- | --- | --- | --- |
|  | **β** | **95% CI** | **P-value** | **β** | **95% CI** | **P-value** |
| **Girls** |  |  |  |  |  |  |
| Time Q14 (ref = Q8) | -0.230 | [-0.276, -0.184] | **<0.001** | -0.230 | [-0.276, -0.184] | **<0.001** |
| Pandemic group (ref = pre-pandemic group) | -0.018 | [-0.059, 0.022] | 0.368 | -0.032 | [-0.072, 0.008] | 0.118 |
| Pandemic group x time (DID) | -0.075 | [-0.136, -0.014] | **0.016** | -0.075 | [-0.135, -0.014] | **0.016** |
| Adjusted R^2^ |  |  | 0.020 |  |  | 0.024 |
| Observations |  |  | 15,058 |  |  | 15,050 |
|  |  |  |  |  |  |  |
| **Boys** |  |  |  |  |  |  |
| Time Q14 (ref = Q8) | -0.325 | [-0.371, -0.278] | **<0.001** | -0.325 | [-0.372, -0.278] | **<0.001** |
| Pandemic group (ref = pre-pandemic group) | 0.003 | [-0.038, 0.044] | 0.874 | -0.019 | [-0.061, 0.022] | 0.354 |
| Pandemic group x time (DID) | 0.040 | [-0.020, 0.101] | 0.190 | 0.040 | [-0.020, 0.101] | 0.192 |
| Adjusted R^2^ |  |  | 0.023 |  |  | 0.031 |
| Observations |  |  | 14,200 |  |  | 14,190 |
|  |  |  |  |  |  |  |

**Note**: Cluster-robust standard errors used to compute 95% confidence intervals. Adjusted model controls for maternal and child covariates (age child at Q14, maternal age, education)

### Number of close friends

**Main results**

| **Friends - standardized** | **Unadjusted model** | | | **Adjusted model** | | |
| --- | --- | --- | --- | --- | --- | --- |
|  | **β** | **95% CI** | **P-value** | **β** | **95% CI** | **P-value** |
| **Girls** |  |  |  |  |  |  |
| Time Q14 (ref = Q8) | -0.148 | [-0.188, -0.108] | **<0.001** | -0.148 | [-0.188, -0.108] | **<0.001** |
| Pandemic group (ref = pre-pandemic group) | 0.019 | [-0.018, 0.055] | 0.323 | 0.009 | [-0.028, 0.046] | 0.626 |
| Pandemic group x time (DID) | -0.195 | [-0.245, -0.145] | **<0.001** | -0.195 | [-0.245, -0.145] | **<0.001** |
| Adjusted R^2^ |  |  | 0.022 |  |  | 0.024 |
| Observations |  |  | 19,536 |  |  | 19,520 |
|  |  |  |  |  |  |  |
| **Boys** |  |  |  |  |  |  |
| Time Q14 (ref = Q8) | -0.235 | [-0.273, -0.197] | **<0.001** | -0.236 | [-0.273, -0.198] | **<0.001** |
| Pandemic group (ref = pre-pandemic group) | 0.002 | [-0.035, 0.038] | 0.917 | -0.003 | [-0.039, 0.035] | 0.881 |
| Pandemic group x time (DID) | -0.049 | [-0.096, -0.001] | **0.044** | -0.049 | [-0.096, -0.001] | **0.044** |
| Adjusted R^2^ |  |  | 0.018 |  |  | 0.018 |
| Observations |  |  | 19,502 |  |  | 19,486 |
|  |  |  |  |  |  |  |

**Note**: Cluster-robust standard errors used to compute 95% confidence intervals. Adjusted model controls for maternal and child covariates (age child at Q14, maternal age, education)

**Sensitivity 1: Including individuals responding before 2022**

| **Friends - standardized** | **Unadjusted model** | | | **Adjusted model** | | |
| --- | --- | --- | --- | --- | --- | --- |
|  | **β** | **95% CI** | **P-value** | **β** | **95% CI** | **P-value** |
| **Girls** |  |  |  |  |  |  |
| Time Q14 (ref = Q8) | -0.149 | [-0.189, -0.109] | **<0.001** | -0.149 | [-0.189, -0.109] | **<0.001** |
| Pandemic group (ref = pre-pandemic group) | 0.010 | [-0.030, 0.050] | 0.632 | 0.002 | [-0.038, 0.042] | 0.920 |
| Pandemic group x time (DID) | -0.183 | [-0.237, -0.128] | **<0.001** | -0.182 | [-0.236, -0.128] | **<0.001** |
| Adjusted R^2^ |  |  | 0.019 |  |  | 0.021 |
| Observations |  |  | 15,484 |  |  | 15,474 |
|  |  |  |  |  |  |  |
| **Boys** |  |  |  |  |  |  |
| Time Q14 (ref = Q8) | -0.238 | [-0.277, -0.200] | **<0.001** | -0.239 | [-0.277, -0.201] | **<0.001** |
| Pandemic group (ref = pre-pandemic group) | 0.020 | [-0.020, 0.059] | 0.329 | 0.016 | [-0.024, 0.056] | 0.428 |
| Pandemic group x time (DID) | -0.034 | [-0.086, 0.018] | 0.206 | -0.034 | [-0.087, 0.018] | 0.196 |
| Adjusted R^2^ |  |  | 0.016 |  |  | 0.017 |
| Observations |  |  | 15,494 |  |  | 15,482 |
|  |  |  |  |  |  |  |

**Note**: Cluster-robust standard errors used to compute 95% confidence intervals. Adjusted model controls for maternal and child covariates (age child at Q14, maternal age, education)

### Overview of results (MoBa)

Overview of MoBa results across main and sensitivity analyses, summarizing direction of Difference-in-difference results. Upward arrow= observed increase during the pandemic; Downward arrow= observed decrease during the pandemic, Dash=no change; Arrows/Dash in brackets = direction of general age trends from 8-14 years. Yellow marking indicates results of sensitivity analyses that differ from main analyses.

## 5. Supplementary results DID analyses DNBC:

### Number of close friends

|  | Unadjusted | | | Adjusted | | |
| --- | --- | --- | --- | --- | --- | --- |
| Term | β | 95% CI | P-value | β | 95% CI | P-value |
| **Girls** |  |  |  |  |  |  |
| Time Q18 (ref = Q11) | 0.162 | [0.140, 0.184] | **<0.001** | 0.162 | [0.140, 0.184] | **<0.001** |
| Pandemic group (ref = pre-pandemic group) | -0.134 | [-0.171, -0.098] | **<0.001** | -0.135 | [-0.171, -0.098] | **<0.001** |
| Pandemic group x time (DID) | 0.136 | [0.087, 0.184] | **<0.001** | 0.135 | [0.087, 0.184] | **<0.001** |
| Adjusted R2 |  |  | 0.011 |  |  | 0.011 |
| Observations |  |  | 34248.000 |  |  | 34216.000 |
| **Boys** |  |  |  |  |  |  |
| Time Q18 (ref = Q11) | 0.448 | [0.422, 0.473] | **<0.001** | 0.448 | [0.422, 0.474] | **<0.001** |
| Pandemic group (ref = pre-pandemic group) | -0.083 | [-0.126, -0.040] | **<0.001** | -0.081 | [-0.124, -0.038] | **<0.001** |
| Pandemic group x time (DID) | 0.075 | [0.019, 0.130] | **0.009** | 0.075 | [0.019, 0.131] | **0.009** |
| Adjusted R2 |  |  | 0.054 |  |  | 0.055 |
| Observations |  |  | 23120.000 |  |  | 23086.000 |

### Self-rated health

|  | Unadjusted | | | Adjusted | | |
| --- | --- | --- | --- | --- | --- | --- |
| Term | β | 95% CI | P-value | β | 95% CI | P-value |
| **Girls** |  |  |  |  |  |  |
| Time Q18 (ref = Q11) | -0.020 | [-0.041, 0.002] | 0.071 | -0.020 | [-0.041, 0.002] | 0.071 |
| Pandemic group (ref = pre-pandemic group) | -0.059 | [-0.092, -0.025] | **<0.001** | -0.055 | [-0.088, -0.022] | **0.001** |
| Pandemic group x time (DID) | 0.008 | [-0.039, 0.055] | 0.733 | 0.009 | [-0.038, 0.055] | 0.720 |
| Adjusted R2 |  |  | 0.000 |  |  | 0.003 |
| Observations |  |  | 34242.000 |  |  | 34210.000 |
| **Boys** |  |  |  |  |  |  |
| Time Q18 (ref = Q11) | -0.154 | [-0.181, -0.128] | **<0.001** | -0.155 | [-0.181, -0.129] | **<0.001** |
| Pandemic group (ref = pre-pandemic group) | -0.037 | [-0.081, 0.006] | 0.089 | -0.035 | [-0.079, 0.008] | 0.109 |
| Pandemic group x time (DID) | 0.054 | [-0.003, 0.111] | 0.065 | 0.054 | [-0.003, 0.111] | 0.062 |
| Adjusted R2 |  |  | 0.005 |  |  | 0.006 |
| Observations |  |  | 23190.000 |  |  | 23156.000 |

### Quality of life

|  | Unadjusted | | | Adjusted | | |
| --- | --- | --- | --- | --- | --- | --- |
| Term | β | 95% CI | P-value | β | 95% CI | P-value |
| **Girls** |  |  |  |  |  |  |
| Time Q18 (ref = Q11) | -0.723 | [-0.742, -0.703] | **<0.001** | -0.722 | [-0.742, -0.703] | **<0.001** |
| Pandemic group (ref = pre-pandemic group) | 0.024 | [-0.007, 0.055] | 0.127 | 0.021 | [-0.010, 0.052] | 0.188 |
| Pandemic group x time (DID) | -0.142 | [-0.185, -0.099] | **<0.001** | -0.142 | [-0.185, -0.100] | **<0.001** |
| Adjusted R2 |  |  | 0.143 |  |  | 0.145 |
| Observations |  |  | 34734.000 |  |  | 34702.000 |
| **Boys** |  |  |  |  |  |  |
| Time Q18 (ref = Q11) | -0.525 | [-0.549, -0.501] | **<0.001** | -0.525 | [-0.550, -0.501] | **<0.001** |
| Pandemic group (ref = pre-pandemic group) | 0.043 | [0.005, 0.081] | **0.028** | 0.040 | [0.002, 0.078] | **0.040** |
| Pandemic group x time (DID) | -0.198 | [-0.253, -0.144] | **<0.001** | -0.199 | [-0.254, -0.145] | **<0.001** |
| Adjusted R2 |  |  | 0.082 |  |  | 0.083 |
| Observations |  |  | 23690.000 |  |  | 23654.000 |

### Sleep

|  | Unadjusted | | | Adjusted | | |
| --- | --- | --- | --- | --- | --- | --- |
| Term | β | 95% CI | P-value | β | 95% CI | P-value |
| **Girls** |  |  |  |  |  |  |
| Time Q18 (ref = Q11) | -1.307 | [-1.323, -1.290] | **<0.001** | -1.306 | [-1.322, -1.289] | **<0.001** |
| Pandemic group (ref = pre-pandemic group) | 0.169 | [0.147, 0.191] | **<0.001** | 0.166 | [0.144, 0.188] | **<0.001** |
| Pandemic group x time (DID) | -0.221 | [-0.257, -0.185] | **<0.001** | -0.222 | [-0.257, -0.186] | **<0.001** |
| Adjusted R2 |  |  | 0.461 |  |  | 0.462 |
| Observations |  |  | 33360.000 |  |  | 33328.000 |
| **Boys** |  |  |  |  |  |  |
| Time Q18 (ref = Q11) | -1.378 | [-1.398, -1.359] | **<0.001** | -1.378 | [-1.397, -1.358] | **<0.001** |
| Pandemic group (ref = pre-pandemic group) | 0.135 | [0.109, 0.162] | **<0.001** | 0.131 | [0.104, 0.157] | **<0.001** |
| Pandemic group x time (DID) | -0.159 | [-0.202, -0.116] | **<0.001** | -0.158 | [-0.201, -0.115] | **<0.001** |
| Adjusted R2 |  |  | 0.500 |  |  | 0.502 |
| Observations |  |  | 22022.000 |  |  | 21994.000 |

### Prosocial behavior

|  | Unadjusted | | | Adjusted | | |
| --- | --- | --- | --- | --- | --- | --- |
| Term | β | 95% CI | P-value | β | 95% CI | P-value |
| **Girls** |  |  |  |  |  |  |
| Time Q18 (ref = Q11) | 0.064 | [0.043, 0.085] | **<0.001** | 0.064 | [0.043, 0.085] | **<0.001** |
| Pandemic group (ref = pre-pandemic group) | 0.027 | [-0.011, 0.065] | 0.163 | 0.022 | [-0.016, 0.060] | 0.255 |
| Pandemic group x time (DID) | 0.007 | [-0.041, 0.055] | 0.775 | 0.007 | [-0.040, 0.055] | 0.758 |
| Adjusted R2 |  |  | 0.001 |  |  | 0.004 |
| Observations |  |  | 32752.000 |  |  | 32724.000 |
| **Boys** |  |  |  |  |  |  |
| Time Q18 (ref = Q11) | 0.009 | [-0.017, 0.035] | 0.508 | 0.009 | [-0.017, 0.035] | 0.508 |
| Pandemic group (ref = pre-pandemic group) | 0.083 | [0.037, 0.128] | **<0.001** | 0.078 | [0.032, 0.123] | **<0.001** |
| Pandemic group x time (DID) | -0.047 | [-0.103, 0.009] | 0.103 | -0.045 | [-0.101, 0.011] | 0.113 |
| Adjusted R2 |  |  | 0.001 |  |  | 0.002 |
| Observations |  |  | 21570.000 |  |  | 21546.000 |

### Internalizing traits

|  | Unadjusted | | | Adjusted | | |
| --- | --- | --- | --- | --- | --- | --- |
| Term | β | 95% CI | P-value | β | 95% CI | P-value |
| **Girls** |  |  |  |  |  |  |
| Time Q18 (ref = Q11) | 0.721 | [0.702, 0.740] | **<0.001** | 0.720 | [0.701, 0.739] | **<0.001** |
| Pandemic group (ref = pre-pandemic group) | -0.042 | [-0.074, -0.009] | **0.011** | -0.036 | [-0.068, -0.004] | **0.027** |
| Pandemic group x time (DID) | 0.069 | [0.027, 0.112] | **0.001** | 0.069 | [0.026, 0.111] | **0.001** |
| Adjusted R2 |  |  | 0.135 |  |  | 0.142 |
| Observations |  |  | 32564 |  |  | 32536 |
| **Boys** |  |  |  |  |  |  |
| Time Q18 (ref = Q11) | 0.221 | [0.196, 0.246] | **<0.001** | 0.221 | [0.196, 0.246] | **<0.001** |
| Pandemic group (ref = pre-pandemic group) | 0.004 | [-0.042, 0.050] | 0.861 | 0.012 | [-0.034, 0.058] | 0.615 |
| Pandemic group x time (DID) | 0.015 | [-0.039, 0.070] | 0.583 | 0.014 | [-0.040, 0.069] | 0.604 |
| Adjusted R2 |  |  | 0.012 |  |  | 0.016 |
| Observations |  |  | 21434.000 |  |  | 21410.000 |

### Externalizing traits

|  | Unadjusted | | | Adjusted | | |
| --- | --- | --- | --- | --- | --- | --- |
| Term | β | 95% CI | P-value | β | 95% CI | P-value |
| **Girls** |  |  |  |  |  |  |
| Time Q18 (ref = Q11) | 0.575 | [0.556, 0.595] | **<0.001** | 0.576 | [0.557, 0.595] | **<0.001** |
| Pandemic group (ref = pre-pandemic group) | -0.036 | [-0.070, -0.001] | **0.043** | -0.027 | [-0.061, 0.008] | 0.129 |
| Pandemic group x time (DID) | 0.044 | [0.002, 0.085] | **0.038** | 0.043 | [0.001, 0.084] | **0.042** |
| Adjusted R2 |  |  | 0.085 |  |  | 0.103 |
| Observations |  |  | 32728.000 |  |  | 32700.000 |
| **Boys** |  |  |  |  |  |  |
| Time Q18 (ref = Q11) | 0.365 | [0.340, 0.389] | **<0.001** | 0.365 | [0.341, 0.390] | **<0.001** |
| Pandemic group (ref = pre-pandemic group) | -0.046 | [-0.093, 0.001] | 0.057 | -0.032 | [-0.079, 0.015] | 0.176 |
| Pandemic group x time (DID) | 0.044 | [-0.010, 0.098] | 0.109 | 0.043 | [-0.011, 0.097] | 0.122 |
| Adjusted R2 |  |  | 0.035 |  |  | 0.048 |
| Observations |  |  | 21550.000 |  |  | 21526.000 |

## 6. References

1	Angold, A., Costello, E. J., Messer, S. C. & Pickles, A. Development of a short questionnaire for use in epidemiological studies of depression in children and adolescents. *International journal of methods in psychiatric research* (1995).

2	Angold, A., Costello, E. J., Pickles, A., Winder, F. & Silver, D. Mood and feelings questionnaire. *Unpublished document. Duke University Developmental Program* (1987).

3	Birmaher, B. *et al.* The screen for child anxiety related emotional disorders (SCARED): Scale construction and psychometric characteristics. *Journal of the American Academy of Child & Adolescent Psychiatry* **36**, 545-553 (1997).

4	Birmaher, B. *et al.* Psychometric properties of the Screen for Child Anxiety Related Emotional Disorders (SCARED): a replication study. *Journal of the American academy of child & adolescent psychiatry* **38**, 1230-1236 (1999).

5	Silva, R. R. *et al.* A rating scale for disruptive behavior disorders, based on the DSM-IV item pool. *Psychiatric Quarterly* **76**, 327-339 (2005).

6	Goodman, A., Lamping, D. L. & Ploubidis, G. B. When to use broader internalising and externalising subscales instead of the hypothesised five subscales on the Strengths and Difficulties Questionnaire (SDQ): data from British parents, teachers and children. *Journal of abnormal child psychology* **38**, 1179-1191 (2010).

7	Goodman, R., Meltzer, H. & Bailey, V. The Strengths and Difficulties Questionnaire: A pilot study on the validity of the self-report version. *European child & adolescent psychiatry* **7**, 125-130 (1998).

8	Goodman, R. Psychometric properties of the strengths and difficulties questionnaire. *Journal of the American Academy of Child & Adolescent Psychiatry* **40**, 1337-1345 (2001).

9	Ware, J. E., Kosinski, M. & Keller, S. D. A 12-Item Short-Form Health Survey: construction of scales and preliminary tests of reliability and validity. *Medical care* **34**, 220-233 (1996).

10	Levin, K. A. & Currie, C. Reliability and validity of an adapted version of the Cantril Ladder for use with adolescent samples. *Social Indicators Research* **119**, 1047-1063 (2014).

11	Hjorth, C. F. *et al.* SEPLINE: Socioeconomic Position in Epidemiological Research—A National Guideline on Danish Registry Data. *Clinical epidemiology*, 593-624 (2025).

# Author contributions

HA, UAV, AS, KL, FF, OBP, OAA, OVE, IOL, KSL, RB obtained funding, designed the study, were responsible for the acquisition of data and for administrative, technical and material support.

HA, JHP, IOL and CM drafted the introduction.

HA and CM drafted the discussion part.

TA and CM supervised the analyses

JHP, NU and IHC analysed the data from MoBa and DNBC and described the results.

MMB and AK were responsible for analysis and reporting of Omtanke2020 data.

LANC, SRO, CE, ES, BAJ, MTB and OBP were responsible for analysis and reporting of DBDS data.

KL, MR and KK were responsible for analysis and reporting of the EstBB data.

AHoffart, SUJ and OVE were responsible for analysis and reporting of the MAP-19 study.

ABN, AS, SHB, KTK, HH, GAN and JAN were responsible for analysis and reporting of the NCC cohort.

SLH was responsible for analysis and reporting of BryDeg2020.

IM, TA, AHauksdottir and ET were responsible for analysis and reporting of the Icelandic C-19 Resilience cohort.

AC and HM were responsible for analysis and reporting of the data from Generation Scotland.

HA, JHP, FF, AL, RER, TX, SL, ISR, KSL, IHC, TA, UAV and OAA interpreted the data and results.

All authors revised the draft critically for important intellectual content.

All authors approved the final version of the paper

All authors agreed to be accountable for all aspects of the work thereby ensuring that questions related to the accuracy or integrity of any part of the work are appropriately investigated and resolved.
